# Unveiling the Over‐Lithiation Behavior of NCM523 Cathode Towards Long‐Life Anode‐Free Li Metal Batteries

**DOI:** 10.1002/advs.202503558

**Published:** 2025-03-20

**Authors:** Ruimin Gao, Minzhi Zhan, Tingcan Li, Pei Xiong, Qian Zhang, Zhefeng Chen, Jike Wang, Xinping Ai, Feng Pan, Liumin Suo, Jiangfeng Qian

**Affiliations:** ^1^ Hubei Key Laboratory of Electrochemical Power Sources College of Chemistry and Molecular Sciences Wuhan University Wuhan Hubei 430072 China; ^2^ School of Advanced Materials Peking University Shenzhen Graduate School Shenzhen 518055 China; ^3^ The Institute for Advanced Studies Wuhan University Wuhan Hubei 430072 China; ^4^ Institute of Physics Chinese Academy of Sciences Beijing National Laboratory for Condensed Matter Physics Beijing 100190 China; ^5^ Center of Materials Science and Optoelectronics Engineering University of Chinese Academy of Sciences Beijing 100049 China

**Keywords:** anode‐free Li metal batteries, Li‐enriched NCM523 cathode, over‐lithiation behavior, structure evolution, valence state evolution

## Abstract

Anode‐free lithium metal batteries (AFLMBs) offer the potential for significantly enhanced energy densities. However, their practical application is limited by a shortened cycling life due to inevitable Li loss from parasitic reactions. This study addresses this challenge by incorporating an over‐lithiated Li_1+_ *_x_* Ni_0.5_Co_0.2_Mn_0.3_O_2_ (Li_1+_ *_x_* NCM523) cathode as an internal Li reservoir to compensate for lithium loss during extended cycling. A rigorous investigation of the deep discharge behavior of the Li_1+_ *_x_* NCM523 cathode reveals a critical over‐lithiation threshold at *x *= 0.7. At this threshold, excess Li^+^ ions are safely accommodated within the crystal structure by a transformation from the LiO_4_ octahedron to two tetrahedral sites. Beyond this threshold (*x* ≥ 0.7), the structural stability of the cathode is significantly compromised due to the irreversible reduction of transition metal (TM) ions. The optimal Li‐rich Li_1.7_NCM523 releases an additional charge capacity of ≈160 mAh g^−1^ during the first charge. Consequently, the AFLMBs (Li_1.7_NCM523||Cu) achieve outstanding capacity retention of 93.3% after 100 cycles at 0.5 C and 78.5% after 200 cycles at 1 C. The findings establish a research paradigm for designing superior over‐lithiated transition metal oxide cathode materials and underscore the critical role of the lithium reservoir in extending the cycle life of AFLMBs.

## Graphical abstract

A comprehensive investigation of the over‐lithiation behavior of Li_1+x_Ni_0.5_Co_0.2_Mn_0.3_O_2_ (Li_1+_ *_x_* NCM523) reveals a critical threshold at *x* = 0.7, beyond which the structural stability of the cathode deteriorates due to irreversible reduction of transition metal ions. The anode‐free Li metal batteries using the optimal Li‐enriched Li_1.7_NCM523 cathode demonstrate remarkable capacity retention of 78.5% after 200 cycles at 1 C, representing the best performance reported to date.

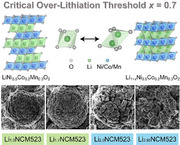

## 1 Introduction

Due to the merits of ultrahigh theoretical capacity (3860 mAh g−¹) and the lowest redox potential (−3.04 V versus SHE), Li metal is regarded as an ideal anode toward energy‐dense lithium metal batteries (LMBs).^[^ ^1^, ^2^ ^]^ Compared to traditional LMBs, anode‐free lithium metal batteries (AFLMBs), which only used a Cu current collector, are poised to reduce the safety hazards of directly handling active Li^0^, simplify the battery production process and further enhance both volumetric and gravimetric energy density.^[^ ^3^, ^4^ ^]^ As a result, AFLMBs attracted significant research interest in recent years. Unfortunately, owing to the poor reversibility of Li plating/stripping as well as the lack of Li compensation, AFLMBs faced substantial challenges related to rapid capacity fading, which hindered their practical application.^[^ ^5^, ^6^ ^]^

Numerous improvement efforts have been proposed, including the development of stable solid‐electrolyte interphases (SEIs) by electrolyte regulation (i.e., high‐concentration,^[^ ^7^, ^8^ ^]^ localized concentration^[^ ^9^, ^10^ ^]^ all‐fluorinated,^[^ ^11^, ^12^ ^]^ and flammable‐retardant electrolytes,^[^ ^13^, ^14^, ^15^, ^16^ ^]^ designing lithophilic substrates to control Li nucleation and growth (i.e., Ag/Au/Sn heterometal‐modified substrates^[^ ^17^, ^18^ ^]^ and N/S/O heteroatom‐doped carbon hosts^[^ ^19^, ^20^ ^]^), rejuvenating dead Li through thermal and chemical activation,^[^ ^21^, ^22^, ^23^ ^]^ and optimized testing protocols like cycling rate and external pressure^[^ ^24^, ^25^ ^]^ to prolong the cycling life of AFLMBs. Despite significant progress, none of these approaches could guarantee 100% cycling efficiency (CE), indicating an inevitable loss of active Li^+^ ions in each cycle.^[^ ^26^, ^27^ ^]^ This gradual accumulation of Li loss during cycling, even if minimal, will eventually lead to the rapid deterioration of AFLMBs.^[^ ^28^, ^29^ ^]^ For instance, a Li plating CE with pretty high values of 99.5 and 99.8% results in a capacity drop to ≈80% after only ≈45 cycles (0.995^^45^) and ≈100 cycles (0.998^^100^), respectively.^[^ ^30^, ^31^ ^]^

Therefore, supplementing the cathode with additional active Li^+^ resources to delay Li exhaustion is crucial for extending the lifespan of AFLMBs.^[^ ^32^, ^33^ ^]^ Li‐containing sacrificial salts, such as Li_2_C_2_O_4_,^[^ ^34^ ^]^ LiNO_3_,^[^ ^35^ ^]^ Li_3_N,^[^ ^36^ ^]^ and Li_2_CO_3_,^[^ ^37^ ^]^ were reported to decompose electrochemically, releasing active Li^+^ ions and significantly boosting lithium reserves with minimal addition (5–10% wt). However, their decomposition required activation at higher voltages beyond the electrolyte's thermodynamic stability window, producing gaseous byproducts, such as CO, CO_2_, or N_2_, which negatively affected the battery's performance.^[^ ^38^, ^39^ ^]^ Alternatively, developing Li‐abundant transition metal oxide cathodes (Li_1+_ *_x_* TMO_2_, where 0 ≤ *x* ≤1, also referred as over‐lithiated or Li‐enriched cathodes)^[^ ^40^, ^41^ ^]^ that can provide extra endogenous Li^+^ ions (beyond its normal stoichiometric ratio of LiTMO_2_) is more favorable for practical applications. This approach (LiTMO_2_ → Li_1+_ *_x_* TMO_2_) leverages a reversible phase transition mechanism, where unoccupied sites in the crystal lattice allowed additional Li^+^ ions to be accommodated without disrupting the overall structure.^[^ ^42^, ^43^ ^]^

For example, Sun et al. achieved electrochemical pre‐lithiation of LiMn_2_O_4_ by over‐discharge to 2.0 V, resulting in the formation of a Li‐enriched Li_1.3_Mn₂O₄.^[^ ^44^ ^]^ The Li_1.3_Mn_2_O_4_ cathode released the extra 0.3 Li^+^ ions during the first charge and cycled stably within the normal potential window of 3.2–4.35 V. An anode‐free cell composed of Cu||Li_1.3_Mn_2_O_4_ outperformed the Cu||LiMn_2_O_4_ control cell by delivering superior capacity retention of 93 versus 25% after 40 cycles. Similarly, Suo et al. synthesized pre‐lithiated Li_1.37_Ni_0.8_Co_0.1_Mn_0.1_O_2_ (Li_1.37_NCM811) by reacting n‐butyl‐Li with NCM811.^[^ ^45^ ^]^ Using this Li_1.37_NCM811 as a cathode, the anode‐free pouch cell achieved an energy density of 447 Wh kg^−1^ and maintained 84% capacity retention after 100 cycles, marking a significant advancement in cycling performance. Nonetheless, the over‐lithiation degree in the reported materials (Li_1.3_Mn_2_O_4_, Li_1.37_NCM811) remained far below their theoretical upper limits (Li_2_Mn_2_O_4_, Li_2_NCM811). The discrepancy between theoretical and practical over‐lithiation behavior was often overlooked and little related research had been conducted. A comprehensive understanding of the structure–performance relationship in over‐lithiated cathodes is helpful to bridge this gap and may open new avenues for developing more Li‐abundant cathodes towards longer‐life AFLMBs.

Herein, we take LiNi_0.5_Co_0.2_Mn_0.3_O_2_ (NCM523) as a model system to investigate its over‐lithiation behavior and related structural/elemental evolution across a wide potential range of 4.3–0.5 V versus Li^+^/Li. A series of pre‐lithiated materials (Li_1.0_NCM523, Li_1.7_NCM523, Li_2.0_NCM523, and Li_3.85_NCM523) with wide variation in lithiation dosage were successfully prepared by electrochemical over‐discharge to 2.5, 1.2, 0.9, and 0.5 V, respectively. We found that appropriate pre‐lithiation enhanced the Li reservoir, whereas excessive pre‐lithiation led to irreversible structural degradation. Upon meticulous evaluation, Li_1.7_NCM523 was identified as the optimal Li‐enriched cathode. When utilized in AFLMBs, Li_1.7_NCM523 provided an additional lithium storage capacity of ≈160 mAh g^−1^, effectively offsetting the inevitable Li loss during cycling and achieving outstanding cycle performance. The Cu||Li_1.7_NCM523 AFLMBs exhibited excellent capacity retention of 93.3% after 100 cycles at 0.5 C and 78.5% after 200 cycles at 1 C, with a more stable Li deposition morphology.

## 2 Results and Discussion

### 2.1 Over‐Lithiation Behavior of Li_1.0_NCM523 Cathode

**Figure 1:**
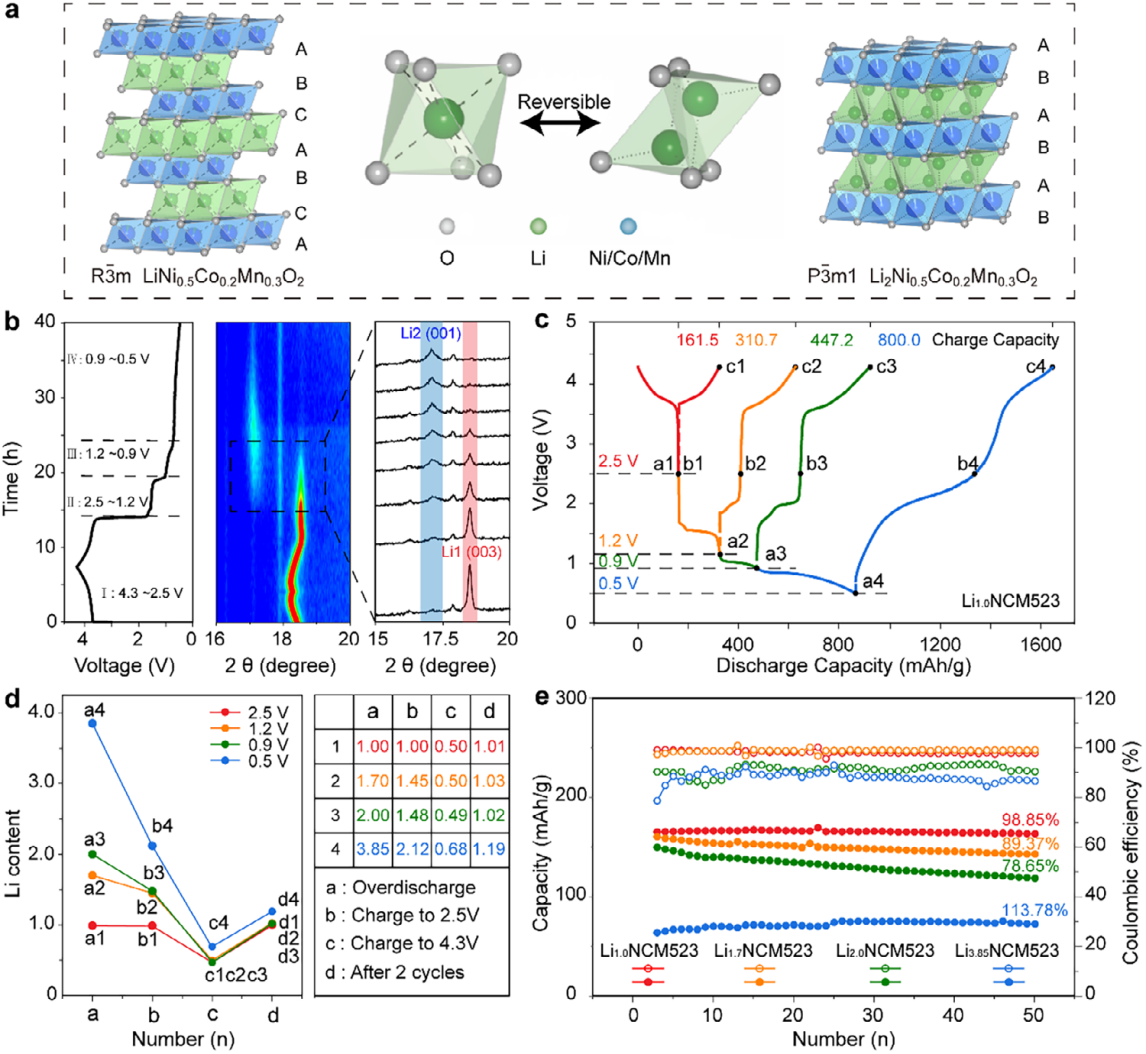
Over‐lithiation Behavior of Li_1+_ *_x_* NCM523 Cathode within a Wide Potential Range of 4.3–0.5 V versus Li^+^/Li. a) Schematic illustration showing the crystal structure transition from Li_1.0_NCM523 to over‐lithiated Li_2.0_NCM523. b) In‐situ XRD patterns of Li_1.0_NCM523 during cycling within normal voltage range of 2.5–4.3 V for the first cycle, followed by over‐discharge to an exceptionally low voltage of 0.5 V. c) The segmented over‐discharge profiles (over‐stoichiometric Li insertion) at different cut‐off voltages of 2.5  (a1), 1.2  (a2), 0.9  (a3), and 0.5 V (a4); followed by subsequent charge profiles (Li extraction) from these over‐lithiated states (a1, a2, a3, a4) through 2.5 V (b1, b2, b3, b4), up to the full charge state at 4.3 V (c1, c2, c3, c4). This test aimed to demonstrate the reversibility of the over‐lithiated process. d) The Li content variations in Li_1.0_NCM523 throughout the processes of electrochemical lithium insertion (a1–a4), lithium extraction (b1–b4, c1–c4), and after two compete charge/discharge cycles (d1–d4), as determined by ICP‐OES. e) Cycling stability of various pre‐lithiated Li_1.0_NCM523, Li_1.7_NCM523, Li_2.0_NCM523 and Li_3.85_NCM523 electrodes at 0.2 C between 2.5–4.3 V. These samples were synthesized via an electrochemical pre‐lithiation method (Figure S2, Supporting Information) by discharging to specific cut‐off voltages of 2.5, 1.2, 0.9, and 0.5 V, respectively (Figure S3, Supporting Information).

Ternary transition metal NCM oxides,^[^ ^46^, ^47^ ^]^ composed of Ni, Co, and Mn elements, represent a promising class of high‐voltage and high‐capacity cathode materials for advanced lithium‐ion batteries. Among various NCM compositions with differing TM ratios, Li_1.0_NCM523 (Li_1.0_Ni_0.5_Co_0.2_Mn_0.3_O_2_) stands out for its balanced overall performance,^[^ ^48^, ^49^ ^]^ making it an ideal model compound for exploring the cathode over‐lithiation mechanism. The pristine Li_1.0_NCM523 (Li1 phase) exhibited a typical layered structure with cubic close packing, crystallizing in the R–3m space group, isostructural to α–NaFeO_2_.^[^ ^50^ ^]^ The Li_1.0_NCM523 can reversibly take up over‐stoichiometric Li^+^ according to Li_1+_ *_x_* NCM523 when it was discharged to below 2.5 V. This pre‐lithiation process propels Li^+^ ions originally located at octahedral sites into tetrahedral positions, as illustrated in **Figure** **1**a.^[^ ^48^ ^]^ This transformation involved a biphasic reaction occurring through displacement of the O–TM–O sheets along the c‐axis.^[^ ^51^ ^]^ Upon full occupation of the tetrahedral sites, the number of Li^+^ ions per unit cell increased from one to two, forming Li_2.0_Ni_0.5_Co_0.2_Mn_0.3_O_2_ (Li_2.0_NCM523, Li2 Phase). Li_2.0_NCM523 retained a layered structure (hexagonal closest packing) with the P–3m1 space group, isostructural to Li_2_NiO_2_ and Ni(OH)_2_.^[^ ^51^ ^]^ However, this model assumes the ideal lithium storage within the cathode but raises questions about whether the actual lattice could accommodate such substantial incorporation of Li ions while maintaining the stability of the crystal framework. Consequently, this work will delve into the over‐lithiation behavior of NCM523 and the structural stability of various pre‐lithiated Li_1+_ *_x_* NCM523.

First, in‐situ X‐ray diffraction (XRD) was used to observe the structural evolution behavior of Li_1.0_NCM523 as it was deeply discharged from 4.3 V to an exceptionally low voltage of 0.5 V versus Li^+^/Li (Figure 1b; Figure S1, Supporting Information). This process can be segmented into four distinct stages.

**Process I (4.3 –2.5 V)**: In this normal charge/discharge voltage range, Li_1.0_NCM523 underwent reversible Li^+^ ions insertion/extraction. During charging, as Li^+^ ions were extracted, the c‐axis first expanded and then gradually contracted, causing the (003) peak of Li_1.0_NCM523 to initially shift to a lower 2θ diffraction angle and then to a higher one.^[^ ^52^, ^53^ ^]^ During discharging, an inverse change in peak positions was observed, with all Bragg diffraction peaks reverting to their original positions, indicating that the cycling process of Li_1.0_NCM523 in the conventional voltage range exhibited good reversibility.^[^ ^54^, ^55^ ^]^ Correspondingly, Li_1.0_NCM523 exhibited a typical solid solution reaction in 4.3 –2.5 V, characterized by a continuous and smooth charge/discharge curve, as shown in Figure 1c (red line). The reaction equation for this process is:

(1) $$\begin{array}{ccc} & & Li_{1-x}Ni_{0.5}Co_{0.2}Mn_{0.3}O_{2}+xLi^{+}+xe^{-}\\ & & =Li_{1.0}Ni_{0.5}Co_{0.2}Mn_{0.3}O_{2}\left(E\approx 3.8Vslope\right) \end{array}$$

**Process II (2.5 –1.2 V)**: Extending the discharge voltage from 2.5  to 1.2 V, a new characteristic peak at 17.35° was observed from in‐situ XRD, corresponding to the (001) plane of Li_2.0_NCM523. This confirmed that Li ions had been intercalated into the cathode material beyond its initial stoichiometric ratio. As over‐discharge progressed, the intensity of the (003) characteristic peak of Li_1.0_NCM523 gradually diminished, while the intensity of the (001) peak of Li_2.0_NCM523 incrementally strengthened, indicating a typical two‐phase lithiation conversion from Li_1.0_NCM523 to Li_2.0_NCM523, referred as the Li1 and Li2 phases, respectively. The discharge profile showed a new plateau at 1.5 V (Figure 1c, orange line). Analyzing the plateau endpoint product by inductively coupled plasma optical emission spectrometer (ICP‐OES) (a2 point, Figure 1c,d) confirmed its elemental ratio consistent with the two‐phase mixture observed in this stage. The reaction equation for this process is:

(2) $$\begin{array}{ccc} & & Li_{1.0}Ni_{0.5}Co_{0.2}Mn_{0.3}O_{2}+0.7Li^{+}+0.7e^{-}\\ & & =Li_{1.7}Ni_{0.5}Co_{0.2}Mn_{0.3}O_{2}\left(E\approx 1.5Vplateau\right) \end{array}$$

**Process III (1.2 –0.9 V)**: In this stage, in‐situ XRD showed that the Li1 phase characteristic peak further weakened while the Li2 phase continued to strengthen. At the cut‐off voltage of 0.9 V, the (003) characteristic peak of the Li1 phase at 18.78° completely disappeared, indicating that this cathode material had fully converted to a single Li2 phase. ICP‐OES also confirmed the fully formation of Li_2.0_NCM523 (a3 point, Figure 1c,d). Another new voltage plateau appeared at 1.0 V from the discharge curve, attributed to stepped over‐lithiation behavior due to increased polarization effects. In original Li_1.0_NCM523, the migration of Li^+^ ions within the Li layer proceeded from one octahedral site to another through a neighboring tetrahedral void.^[^ ^56^ ^]^ Given that the involved tetrahedral vacancy shares a face with three octahedral within the Li layer, the migration of Li^+^ ions within the Li_1.0_NCM523 structure exhibits a relatively low energy barrier.^[^ ^57^, ^58^ ^]^ However, as pre‐lithiation progressed, the tetrahedral vacancies became increasingly occupied, which inevitably impeded the diffusion pathways of Li^+^ ions. The reaction equation for this stage is:

(3) $$\begin{array}{ccc} & & Li_{1.7}Ni_{0.5}Co_{0.2}Mn_{0.3}O_{2}+0.3Li^{+}+0.3e^{-}\\ & & =Li_{2.0}Ni_{0.5}Co_{0.2}Mn_{0.3}O_{2}\left(E\approx 0.9Vplateau\right) \end{array}$$

**Process IV (0.9–0.5** **V)**: Finally, during deep discharge to 0.5 V, a significant decrease in the intensity of the Li2 phase (001) characteristic peaks was observed in the in‐situ XRD. This was due to the cathode material undergoing excessive reduction, leading to irreversible structural damage and transitioning from crystalline to amorphous states.^[^ ^59^, ^60^ ^]^ The charge/discharge curve exhibited an abnormal “slope” pattern (Figure 1c, blue line). Additionally, the ICP‐OES indicated an unusually high Li content of 3.85 at the discharge endpoint (due to the decomposition product of Li_2_O) (a4 point, Figure 1d), indicating the displacement reaction resulted in severe degradation of Li_2_NCM523 into TM^0^ and Li_2_O. The reaction equation for this stage is:

(4) $$\begin{array}{ccc} & & Li_{2.0}Ni_{0.5}Co_{0.2}Mn_{0.3}O_{2}+nLi^{+}+ne^{-}\\ & & =Ni^{0}/Co^{0}/Mn^{0}\cdot metals+Li_{2}O\;\left(E\approx 0.5V\cdot plateau\right) \end{array}$$

Therefore, we identified 2.5, 1.2, 0.9, and 0.5 V as specific nodes in the structural transformation of Li_1.0_NCM523 during over‐discharge, corresponding respectively to the occurrence of Li_1.0_NCM523 (single Li1 phase), Li_1.7_NCM523 (Li1 and Li2 biphasic co‐existence), Li_2.0_NCM523 (single Li2 phase), and Li_3.85_NCM523 (amorphous phase, decomposition).

For AFLMBs applications, one critical question was whether the pre‐intercalated Li^+^ ions could be reversibly extracted for Li compensation. Figures 1c and S3 (Supporting Information) show that each over‐discharge plateau had a counterpart charge plateau. For instance, Li_1.7_NCM523 exhibited a charge plateau at 1.8 V (with a charge capacity of 80 mAh g^−1^) corresponding to the over‐discharge plateau at 1.5 V (with a discharge capacity of 150 mAh g^−1^). Li_2.0_NCM523 displayed two charge plateaus at 1.8  and 2.0 V (180 mAh g^−1^), corresponding to over‐discharge plateaus at 1.5  and 1.0 V (345 mAh g^−1^). The additional small discharge plateau at 1.65 V corresponded to the SEI layer formation (Figure S4, Supporting Information). Crucially, the amount of Li extracted was significantly less than the amount intercalated, indicating that only a portion of the inserted Li^+^ ions could be extracted at low voltages. ICP‐OES analysis showed that the Li contents of these intermediates were consistently 1.45 after charging back to 2.5 V (b2,b3 points, Figure 1c,d).

Providentially, the remaining Li^+^ ions could be extracted entirely in the high voltage range (above 4.0 V). Li_1.7_NCM523 and Li_2.0_NCM523 exhibited total charge capacities of 335 and 470.3 mAh g^−1^, respectively, which were very close to their total discharge capacities of 328 and 475 mAh g^−1^. ICP‐OES also demonstrated that Li_1.0_NCM523, Li_1.7_NCM523, and Li_2.0_NCM523 all converted to the same end‐product (Li_0.5_NCM523) upon full charging to 4.3 V (c1, c2, and c3 points, Figure 1c,d), thereby confirming the reversibility of the pre‐lithiation process. Such a huge voltage hysteresis to extract the residual Li^+^ ions was probably caused by the structural degradation generated during the over‐discharge process, which blocked Li^+^ ions diffusion channels and hampered the redox reaction of TM ions, as demonstrated by the galvanostatic intermittent titration test (GITT) (Figure S5, Supporting Information) and the electrochemical impedance spectroscopy (EIS) test (Figure S6, Supporting Information). After two complete cycles, the Li content (d1–d3 points, Figure 1d) of Li_1.7_NCM523 and Li_2.0_NCM523 was entirely consistent with the original Li_1.0_NCM523. In stark contrast, Li_3.85_NCM523 showed significant abnormalities due to irreversible decomposition (b4, c4, and d4 points, Figure 1d).

Another concern was the impact of the over‐lithiation process on the cycling stability of Li_1+_ *_x_* NCM523. Therefore, Li_1.7_NCM523, Li_2.0_NCM523, and Li_3.85_NCM523 were assembled into Li||Li_1+_ *_x_* NCM523 half‐cells (Figure 1e). Using a ternary electrolyte and cycling in a normal voltage range of 2.5‐4.3 V, all Li||Li_1+_ *_x_* NCM523 exhibited a deteriorated initial discharge capacity and capacity retention compared to primary Li||Li_1.0_NCM523 (163.8 mAh g^−1^, 98.85% after 50 cycles). Li||Li_1.7_NCM523 slightly decreased to 160.4 mAh g^−1^ with a retention rate of 91.53%, and Li||Li_2.0_NCM523 significantly dropped to 151.5 mAh g^−1^ and 83.27%. In particular, Li||Li_3.85_NCM523 only had an extremely low initial discharge capacity of 60 mAh g^−1^, indicating serious decomposition. The huge irreversible capacity renders Li_3.85_NCM523 non‐competitive. Based on the above analysis, we can preliminarily identify Li_1.7_NCM523 as the optimal over‐lithiated cathode. In the following sections, we will further validate this conclusion through structural characterization, surface chemistry analysis, and density functional theory (DFT) calculation.

### 2.2 Morphology and Phase Structure Evolution in Li_1+_ *_x_* NCM523 Cathode

**Figure 2:**
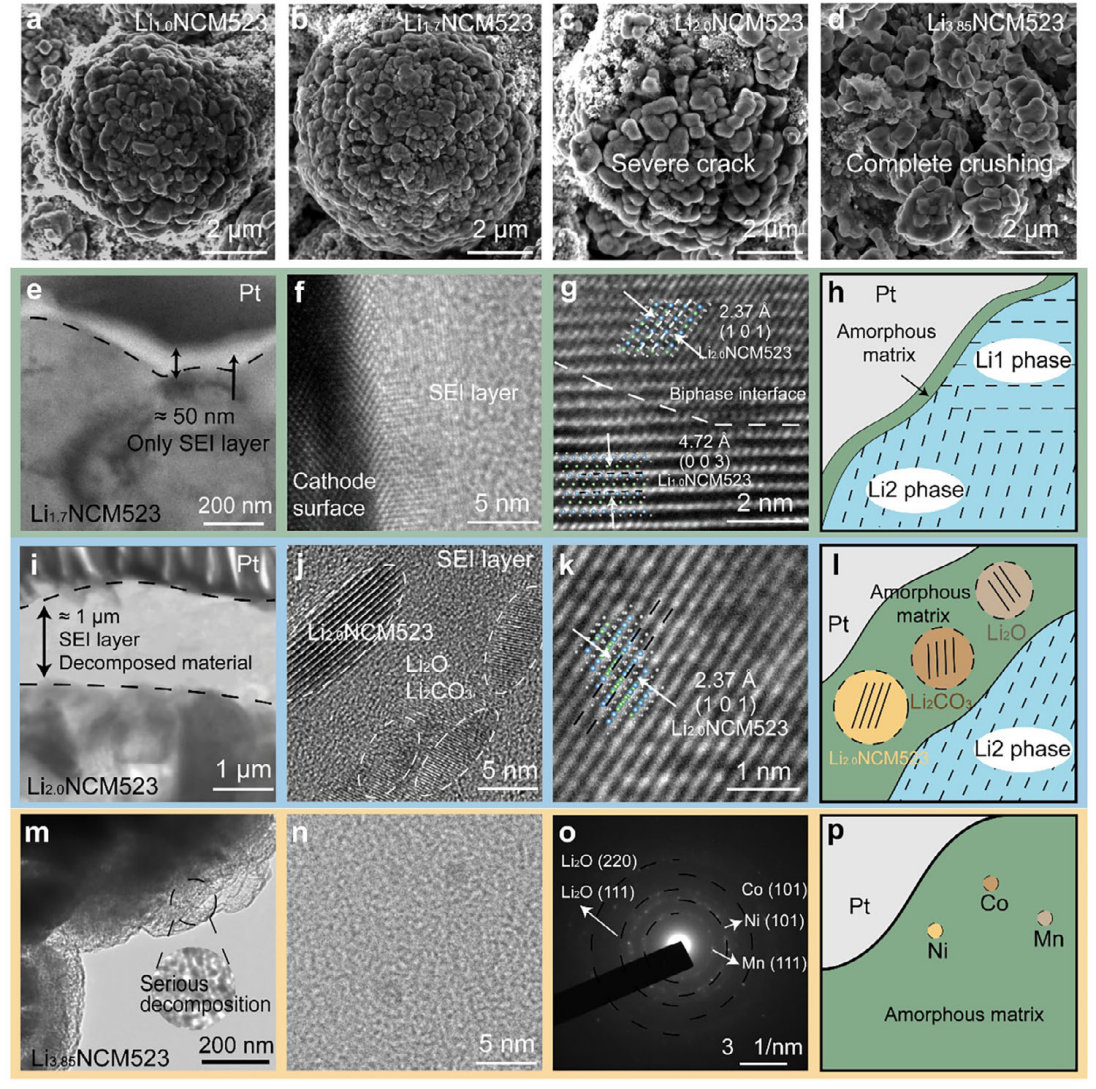
Morphology and Phase Structure Evolution in the Over‐lithiated Li_1+_ *_x_* NCM523 Cathode. a–d) SEM images of Li_1.0_NCM523, Li_1.7_NCM523, Li_2.0_NCM523, and Li_3.85_NCM523 samples, illustrating the progressive fracturing of Li_1+_ *_x_* NCM523 particles with increased lithiation degree. e–g) HR‐TEM images of Li_1.7_NCM523 sample in different regions: e) interface between the SEI layer and Li_1.7_NCM523; f) the thin SEI layer; g) Li_1.7_NCM523 lattice, showing the co‐existence of Li1 phase and Li2 phase. i–k) HR‐TEM images of Li_2.0_NCM523 sample in different regions: i) interface between the surficial layer and Li_2.0_NCM523; j) the thick surficial layer; k) Li_2.0_NCM523 lattice, displaying a single Li2 phase. m,n) HR‐TEM images of Li_3.85_NCM523 sample. o) The SAED of Li_3.85_NCM523, showing its amorphous state and decomposition. h, l, p) Schematic diagrams illustrating the microstructure of h) Li_1.7_NCM523; l) Li_2.0_NCM523; p) Li_3.85_NCM523.

Scanning electron microscopy (SEM) was employed to examine the particle morphology of Li_1.0_NCM523, Li_1.7_NCM523, Li_2.0_NCM523, and Li_3.85_NCM523 (**Figure** **2**a,d). The particle structure of Li_1.7_NCM523 remained intact and dense (Figure 2b), resembling that of Li_1.0_NCM523 (Figure 2a). However, when the lithiation degree was excessive, primary particle deformation and grain boundaries occurred (Figure 2c,d), resulting in severe cracks and large voids in the secondary particles.^[^ ^61^ ^]^ The Li_2.0_NCM523 exhibited significant particle cracking, and Li_3.85_NCM523 was wholly pulverized, caused by the anisotropic lattice volume expansion due to the excess insertion of Li^+^ ions.^[^ ^62^ ^]^ During cycling, these structural deteriorations increased the contact area between the cathode material and electrolyte, leading to severe side reactions and undesirable cycling stability.^[^ ^63^, ^64^ ^]^

High‐resolution transmission electron microscopy (HR‐TEM) provided further insight into the microstructure of Li_1+_ *_x_* NCM523 samples. First, a significant change in the surface film was observed. The surficial layers on Li_1.0_NCM523 (Figure S7b, Supporting Information) and Li_1.7_NCM523 (Figure 2e,f; Figure S8b, Supporting Information) were thin (≈50 nm) and composed of amorphous organic species formed from the electrolyte reduction during the over‐discharge process. In contrast, Li_2.0_NCM523 exhibited a much thicker surface film (≈1 µm, Figure 2i), consisting of nanocrystalline layered oxides, Li_2_O, etc. This suggests that, in addition to the secondary particle fracturing observed in Figure 2c, Li_2.0_NCM523 also underwent a sort of interfacial structure degradation during the over‐lithiation process.^[^ ^18^ ^]^ These deteriorated structures might be the root cause of the inferior electrochemical performance Li_2.0_NCM523 compared to Li_1.7_NCM523 (Figure 1e).

Additionally, observations of the internal crystal structure revealed that Li_1.0_NCM523 exhibited a homogenous Li1 phase (Figure S7a,c, Supporting Information). In contrast, Li_1.7_NCM523 displayed a biphasic structure (Figure 2g), with a distinct interface between the Li1 phase and Li2 phases, characterized by (003) (d = 4.72 Å) and (101) (d = 2.37 Å) planes (Figure 2g; Figure S8c,d, Supporting Information), consistent with the XRD results (Figure 1b; Figure S9, Supporting Information).^[^ ^52^, ^65^ ^]^ Notably, time‐of‐flight secondary ion mass spectrometry (TOF‐SIMS) confirmed the homogeneity of lithium insertion, with no Li gradient present from the interior to the surface of Li_1.7_NCM523 particles (Figure S10, Supporting Information). Upon further reduction to Li_2.0_NCM523, the internal structure transformed to a single Li2 phase (Figure 2k; Figure S11b, Supporting Information). In Li_3.85_NCM523, the deep‐discharging severely disrupted the crystalline structure, resulting in a fully amorphous state (Figure 2m,n). Selected area electron diffraction (SAED) revealed diffraction rings corresponding to Ni^0^, Co^0^, Mn^0^ metals and Li_2_O (Figure 2o), which directly confirmed that Li_3.85_NCM523 had undergone severe decomposition reactions. The schematic diagrams in Figure 2h,l,p vividly illustrate the microstructure of Li_1.7_NCM523, Li_2.0_NCM523, and Li_3.85_NCM523, respectively.

### 2.3 Elemental Valence State Evolution in Li_1+_ *_x_* NCM523 Cathodes

**Figure 3:**
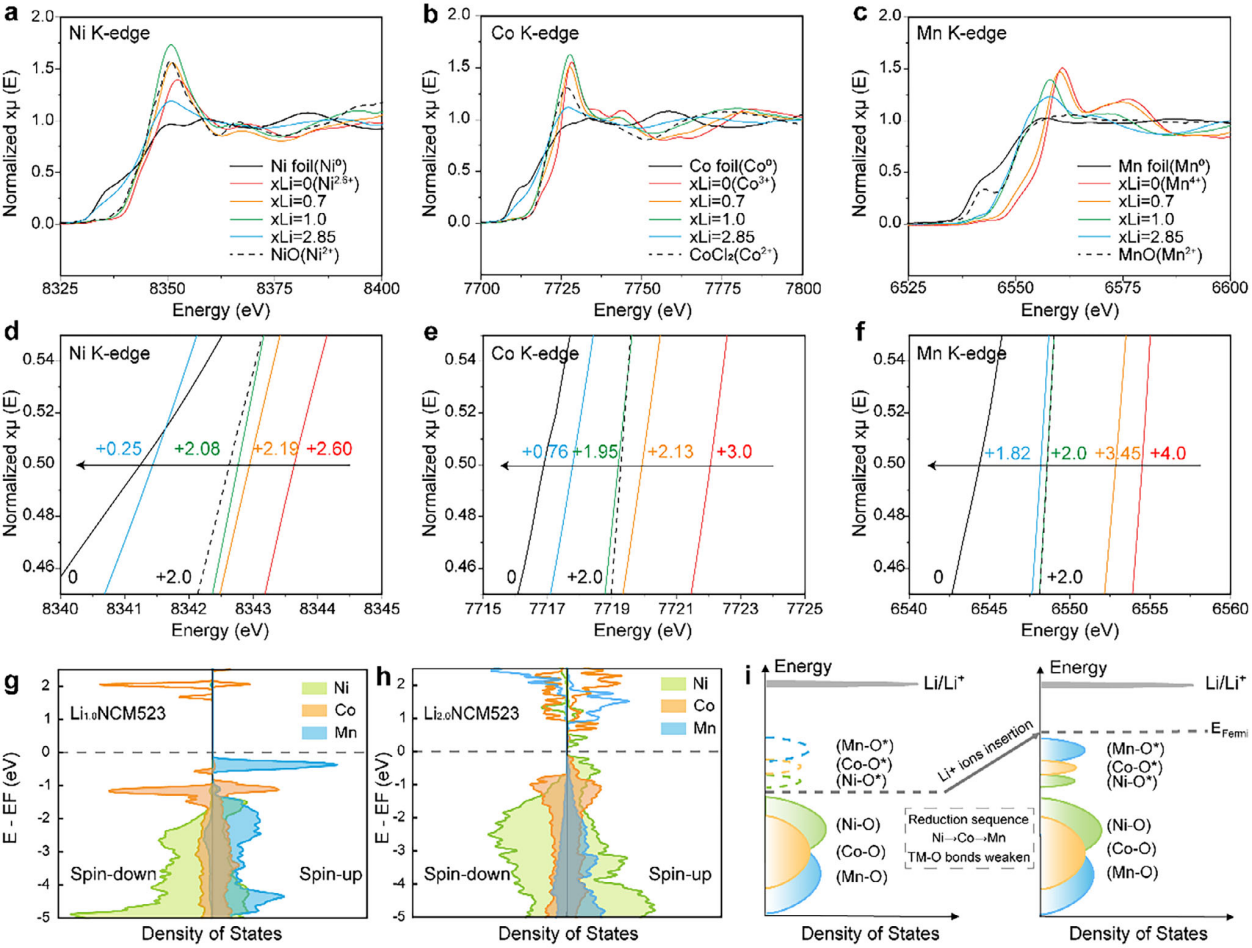
The Bulk Oxidation State and Reduction Sequence of TM elements in Li_1+_ *_x_* NCM523 Cathodes. a) Ni K‐edge, b) Co K‐edge, and c) Mn K‐edge XANES and their corresponding local magnified images of d) Ni (8340–8345 eV), e) Co (7715–7725 eV), and f) Mn (6540–6560 eV) in Li_1+_ *_x_* NCM523 with *x *= 0, 0.7, 1.0, and 2.85. Pristine Li_1.0_NCM523 (Ni^2.60+^, Co^3.0+^, Mn^4.0+^), NiO (Ni^2+^), CoCl_2_ (Co^2+^), and MnO (Mn^2+^) are used as standards. Calculated density of states (DOS) of g) Li_1.0_NCM523 and h) Li_2.0_NCM523. i) The scheme of DOS changes during the reduction process from Li_1.0_NCM523 to Li_2.0_NCM523.

Hard X‐ray absorption spectroscopy (XAS) measurements has a high penetration depth, making it well‐suited for analyzing bulk materials and studying their internal electronic structure and coordination environment. XAS were performed on the prepared Li‐enriched Li_1+_ *_x_* NCM523 materials at different lithiation states, including Li_1.0_NCM523 (pristine material, *x *= 0), Li_1.7_NCM523 (1.2 V, *x *= 0.7), Li_2.0_NCM523 (0.9 V, *x *= 1.0), and Li_3.85_NCM523 (0.5 V, *x *= 2.85). **Figure** **3** shows the normalized (3a) Ni K‐edge, (3b) Co K‐edge, and (3c) Mn K‐edge X‐ray absorption near edge structure (XANES) of Li_1+_ *_x_* NCM523. The oxidation state of each TM element  is estimated from the standard spectra using a linear relationship between the edge energy at a normalized intensity of 0.5 and the oxidation state, as reported in previous studies.^[^ ^66^, ^67^, ^68^ ^]^ The standards used are NiO (Ni^2+^), CoCl_2_ (Co^2+^), MnO (Mn^2+^), and the pristine Li_1.0_NCM523 (Ni^2.6+^, Co^3+^, Mn^4+^) (Figure 3d–f, red line).^[^ ^69^, ^70^ ^]^

Overall, as the degree of lithiation increases, the absorption edge positions of all three TM elements shift to lower energy, indicating a continuous decrease in their valence state. For Li_1.7_NCM523, a significant shift in the Ni K‐edge position was observed, indicating a reduction in the bulk oxidation state of Ni from Ni^2.6+^ to Ni^2.19+^ (Figure 3d, orange line). Correspondingly, the bulk oxidation states of Co (Figure 3e, orange line) and Mn (Figure 3f, orange line) decreased from Co^3^⁺ and Mn⁴⁺ to Co^2.13+^ and Mn^3.45+^, respectively. This shows that during this stage, three elements Ni, Co, and Mn, all participate in the reduction process, with Ni and Co being the dominant contributors. Mn undergoes only partial reduction to Mn^3.45+^, indicating the coexistence of Mn^2+^ and Mn^4+^, with Mn^4+^ being the predominant species. In Li_2.0_NCM523, the bulk oxidation states of the TM further decreased to Ni^2.08+^ (Figure 3d, green line), Co^1.95+^ (Figure 3e, green line), and Mn^2+^ (Figure 3f, green line). Compared to Li_1.7_NCM523, Ni and Co in Li_2.0_NCM523showed only slight reductions, while Mn exhibited a more significant change, with all Mn^4+^ fully reduced to Mn^2+^. This indicates that the reduction at this stage primarily involves Mn element. As the lithiation progressed further to Li_3.85_NCM523, the bulk oxidation states of Ni and Co dropped drastically to Ni^0.25+^ (Figure 3d, blue line) and Co^0.76+^ (Figure 3e, blue line), while Mn decreased to Mn^1.82+^ (Figure 3f, blue line). The result gives that Li_3.85_NCM523 has irreversible decomposition due to over‐reduction, leading to the presence of TM simple substance (Ni^0^, Co^0^, and Mn^0^).

The XAS results reveal that during the reduction from Li_1.0_NCM523 to Li_1.7_NCM523, the process is dominated by the reduction of Ni and Co. And the further reduction from Li_1.7_NCM523 to Li_2.0_NCM523 primarily involves the reduction of Mn. Therefore, we speculate that during the over‐lithiation process, the reduction of Ni and Co elements occurs first, followed by the reduction of Mn. To validate this hypothesis, we conducted DFT calculations to understand the reduction sequence and valence change of TM elements in the over‐lithiation procedure. The calculated Density of states (DOS) for Li_1.0_NCM523 and Li_2.0_NCM523 (Figure 3g,h) describe the bonding environments and redox activity of TM cations. In Li_1.0_NCM523, unoccupied Ni^3+^, Co^3+^, and Mn^4+^ states are sequentially located above the conduction band minimum (CBM). After lithiation to Li_2.0_NCM523, the Fermi level rises, and electrons fill these empty bands. The reduction of Ni^3+^ to Ni^2+^ and Co^3+^ to Co^2+^ takes priority over the reduction of Mn^4+^ to Mn^2+^. This confirms the hypothesis derived from the XAS data that the reduction sequence in over‐lithiated NCM523 follows the order Ni → Co → Mn.

**Figure 4:**
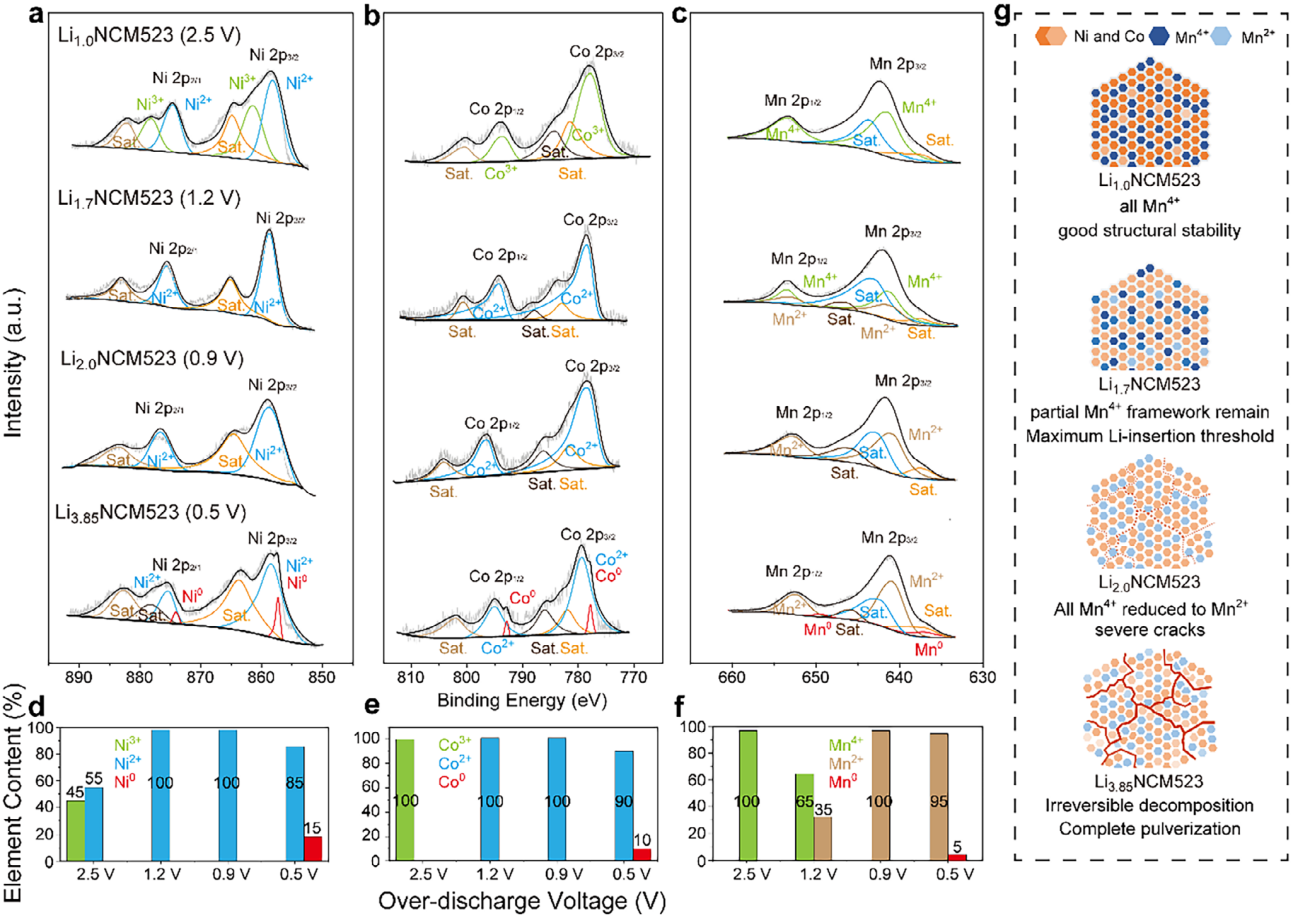
Elemental Valence State Evolution in Li_1+_ *_x_* NCM523 Cathodes. a–c) XPS spectra of Li_1.0_NCM523, Li_1.7_NCM523, Li_2.0_NCM523, and Li_3.85_NCM523 electrodes, consisting of a) Ni 2p, b) Co 2p, and c) Mn 2p spectra. d–f) The ratio variation of different valence states of d) Ni^3+^/Ni^2+^/Ni^0^, e) Co^3+^/Co^2+^/Co^0^, and f) Mn^4+^/Mn^2+^/Mn^0^ during the over‐lithiation process. Before conducting XPS analysis, Ar⁺ etching was performed for 180 s to completely remove interference from the surface layer. g) Schematic diagram of macroscopic morphology changes with variation in Mn element valence states of Li_1+_ *_x_* NCM523 during over‐discharging.

X‐ray photoelectron spectroscopy (XPS), which focuses on the chemical composition, elemental valence states, and electronic states of material surfaces with higher resolution, is employed to further analyze the reduction trends and average valence states of TM elements in Li_1+_ *_x_* NCM523. XPS serves as a further verification and supplement to the conclusions drawn from XAS, with both exhibiting strong consistency (Figure S12, Supporting Information). In pristine Li_1.0_NCM523, Ni^3^⁺ (862.5 eV/878.2 eV) and Ni^2^⁺ (858.1 eV/876.3 eV) coexisted, with a molar ratio of Ni^3+^: Ni^2+^ ≈ 55: 45%,^[^ ^71^, ^72^ ^]^ and Co existed solely as Co^3+^. In Li_1.7_NCM523 and Li_2.0_NCM523, all Ni^3+^ and Co^3+^ was reduced to Ni^2+^ and Co^2+^, respectively.^[^ ^72^ ^]^ Upon further discharge to Li_3.85_NCM523, metallic Ni^0^ and Co^0^ was observed, indicating that a decomposition reaction had occurred, as showed in **Figure** **4**a,d (Ni) and Figure 4b,e (Co).

For the Li_1.0_NCM523 cathode, the Mn element exists in the +4 state (Figure 4c), which is well‐known for its crucial role in maintaining structural stability.^[^ ^73^ ^]^ Mn^4+^ has high redox potential and electrochemical inertia, thus it serves as a “framework” that mitigates the volumetric changes throughout the normal cycling process.^[^ ^74^, ^75^, ^76^ ^]^ Upon lithiation to Li_1.7_NCM523, a portion of Mn^4+^ was reduced to Mn^2+^, with a ratio of Mn^4+^: Mn^2+^ ≈ 65: 35% (Figure 4f). The remaining Mn^4+^ continued to provide skeletal support to the cathode structure. However, in Li_2.0_NCM523 and Li_3.85_NCM523, only Mn^2^⁺ and even Mn^0^ were observed, along with particle fragmentation of these two samples (Figure 4c). Based on these findings, we infer that during deep lithiation, the involvement of all Mn^4+^ in reduction reactions leads to the loss of its role in stabilizing the crystal structure, ultimately resulting in cracking and fracturing of cathode particle (Figure 4g).^[^ ^77^, ^78^ ^]^ In addition, DFT calculations showed that when all TM elements are reduced to the +2 state, the antibonding states of TM–O bonds are filled, causing bond strength weakens, thus leading to the instability in the crystal lattice and primary particles (Figures S13,S14, Supporting Information). This explains the deterioration in structural stability and cycling performance of over‐lithiated Li_2.0_NCM523 and Li_3.85_NCM523.

Therefore, it is essential to avoid blindly pursuing excessive pre‐lithiation. *x* = 0.7 represents the maximum tolerable lithiation threshold for Li_1+_ *_x_* NCM523, the optimal over‐lithiated point to balance storing more active Li^+^ ions and preserving the structure stability.

### 2.4 Electrochemical Performance of Cu||Li_1+_ *_x_* NCM523 Cells

**Figure 5:**
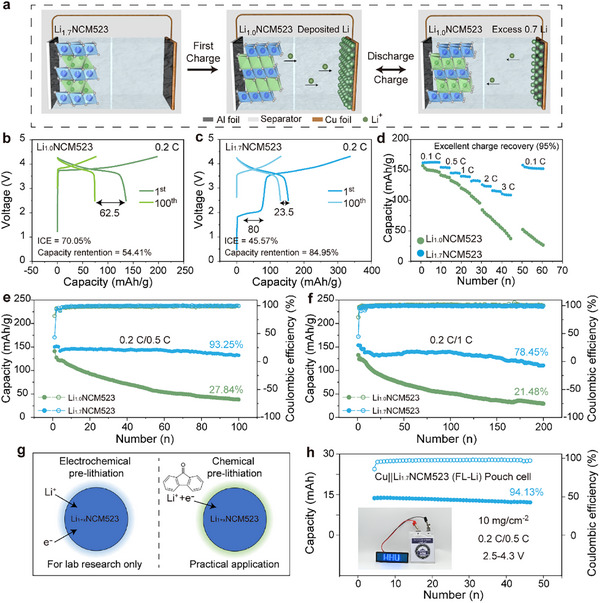
Electrochemical Performance of Cu||Li_1.7_NCM523 Anode‐free Cells. a) Schematic illustration of the working mechanism of Cu||Li_1.7_NCM523 cells, demonstrating how Li‐enriched Li_1.7_NCM523 cathode extends the lifespan of AFLMBs. The voltage/capacity profiles of b) Cu||Li_1.0_NCM523 and c) Cu||Li_1.7_NCM523. d) The rate performance of Cu||Li_1.0_NCM523 and Cu||Li_1.7_NCM523. Cycling performances of Cu||Li_1.0_NCM523 and Cu||Li_1.7_NCM523 e) at 0.2 C (charge)/0.5 C (discharge) after 100 cycles and f) at 0.2 C (charge)/1 C (discharge) after 200 cycles in 2.5–4.3 V. The half‐cell performance of Li||Li_1.0_NCM523 and Li||Li_1.7_NCM523 as showed in Figures S15,S16 (Supporting Information). g) Schematic of the lab‐scale electrochemical pre‐lithiation (left) and practical chemically pre‐lithiation (right, using 9‐fluorenone‐lithium as the lithiation reagent). h) The cycling stability and coulombic efficiency of the Cu||Li_1.7_NCM523 (FL‐Li/THF) anode‐free pouch cell, and a photograph of this anode‐free pouch cell in working.

In AFLMBs, active Li solely comes from the cathode and is inherently limited. Therefore, using Li‐enriched cathode materials to provide extra Li reserves effectively improves battery lifespan. **Figure** **5**a outlines the operating mechanism of pre‐lithiated Li_1.7_NCM523 in AFLMBs. During the initial charge, intrinsic and pre‐intercalated Li^+^ ions are extracted from Li_1.7_NCM523 and transported to the Cu current collector. Upon discharging, the stoichiometric Li^+^ ions return to the cathode, restoring to their original Li_1.0_NCM523 form for subsequent cycles. The extra 0.7 Li remained on the Cu current collector, acting as a reservoir to compensate for the Li loss, thereby improving the cycling stability of the AFLMBs.

Figure 5b,c present the charge/discharge profiles for Cu||Li_1.0_NCM523 and Cu||Li_1.7_NCM523 at 0.2 C, respectively. The similar reversible capacities observed in both cells suggested that the intercalated Li^+^ ions could be fully extracted and that the pre‐lithiation process is reversible. After 100 cycles, the capacity retention for Cu||Li_1.7_NCM523 was significantly high at 85.0%, in stark contrast to the 54.4% for Cu||Li_1.0_NCM523, which experienced pronounced capacity fading after 20 cycles (Figure S17, Supporting Information). The abundant Li reserves in Li_1.7_NCM523 effectively replenished the Li loss, significantly enhancing cycle stability. Additionally, the dQ/dV test (Figure S18, Supporting Information) showed that Li_1.7_NCM523 and Li_1.0_NCM523 exhibited analogous phase transitions except for two additional Li extraction characteristic peaks at 2.0  and 3.5 V, confirming that the pre‐lithiation process did not damage the cathode's structural stability.^[^ ^79^ ^]^

Cu||Li_1.7_NCM523 also exhibited excellent rate performance, particularly at high‐rate conditions (Figure 5d). At 3 C, Cu||Li_1.7_NCM523 achieved a reversible capacity of 108.5 mAh g^−1^, significantly outperforming the 51.3 mAh g^−1^ of Cu||Li_1.0_NCM523 (Figures S19,S20, Supporting Information). When returned to 0.1 C, the capacity of Cu||Li_1.7_NCM523 recovered to 151.8 mAh g^−1^, retaining 95% of its initial capacity, while Cu||Li_1.0_NCM523 suffered irreversible lithium depletion under similar conditions.

Figure 5e,f compare the long‐term cycling performance of Cu||Li_1.0_NCM523 and Cu||Li_1.7_NCM523 cells. Evidently, Cu||Li_1.7_NCM523 displayed a remarkable enhancement in cyclic stability. After 100 cycles at 0.5 C, the capacity retention of Cu||Li_1.7_NCM523 was 93.3%, in stark contrast to Cu||Li_1.0_NCM523, which only maintained 27.8% at the same test conditions. At a more demanding rate of 1 C, Cu||Li_1.7_NCM523 sustained a remarkable capacity retention of 95.3% after 100 cycles and 78.5% even after 200 cycles, while Cu||Li_1.0_NCM523 dropped below 50% after just 50 cycles (Figures S21,S22, Supporting Information). The cycling performance of Cu||Li_1.7_NCM523 surpassed all previously reported achievements in the current literature (Figure S23, Supporting Information), clearly validating the significant role of the Li‐enriched cathode.

The electrochemical prelithiation method involves a complex process on cell assembly/disassembly, making it suitable only for small‐scale laboratory research, and not capable for mass or industrial production (Figure 5g). Therefore, we propose a chemical lithiation method using 9‐fluorenone‐lithium (FL‐Li) reagent^[^ ^80^ ^]^ for the scalable preparation of the Li₁.₇NCM523 electrode. At 0.5 C rate, Cu||Li_1.7_NCM523 (FL‐Li) retains the capacity retention of 94.13% after 50 cycles (Figure 5h; Figure S24, Supporting Information). In addition, we also assembled Cu||Li_1.7_NCM523 with a non‐flammable triethyl phosphate‐based electrolyte^[^ ^16^ ^]^ (Figure S25, Supporting Information), further enhancing the safety. All these results indicate that the Li‐enriched Li_1.7_NCM523 has significant practical application potential in the anode‐free cells.

### 2.5 Evolution of Li Plating Morphology During Cycling

**Figure 6:**
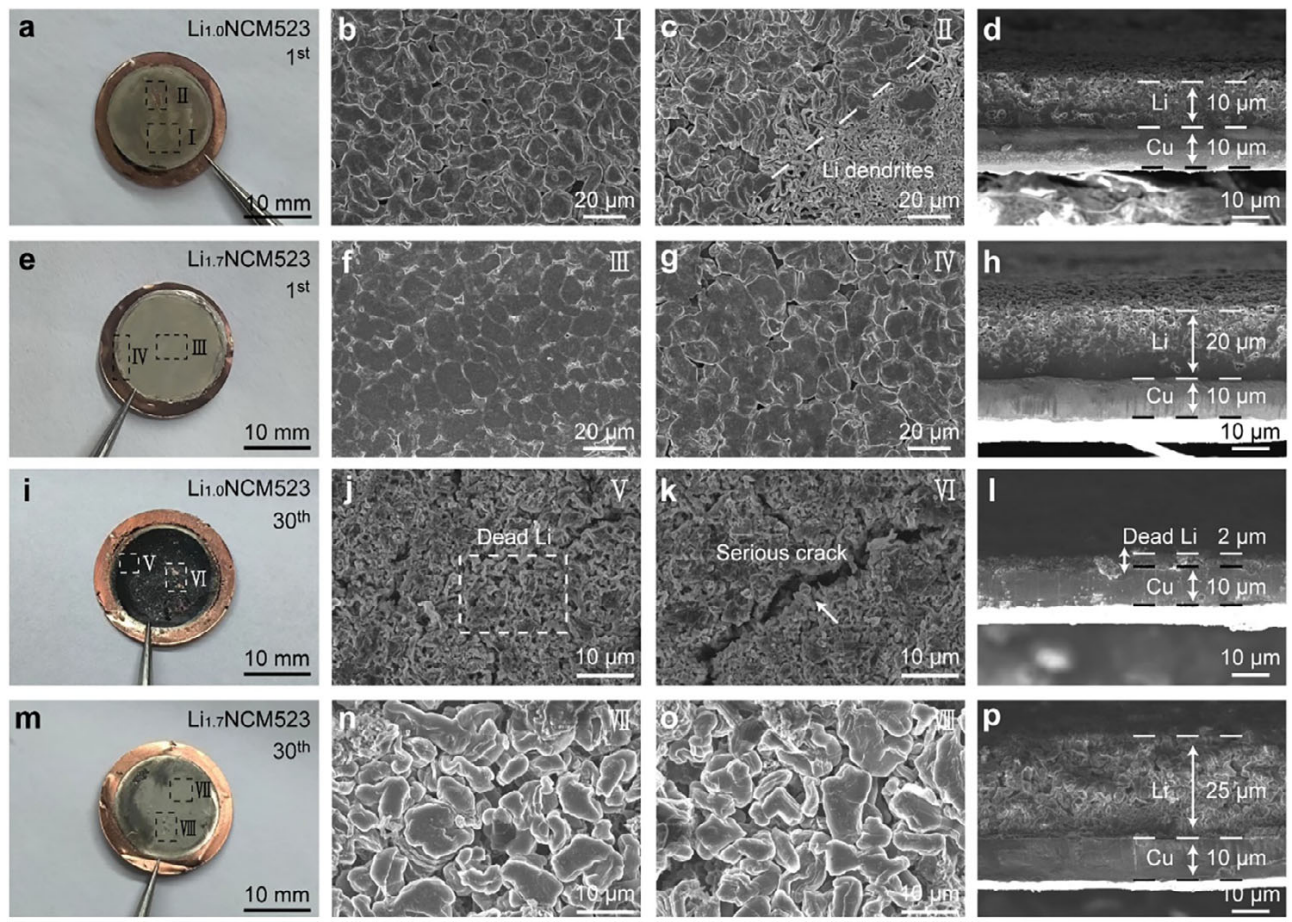
Morphological Characterizations of the Fully Plated Li on Cu Current Collectors. a–d) Cu||Li_1.0_NCM523 cell after 1st charge, e–h) Cu||Li_1.7_NCM523 cell after 1st charge, i–l) Cu||Li_1.0_NCM523 cell after 30^th^ cycles, and m–p) Cu||Li_1.0_NCM523 cell after 30th cycles, respectively. The first column shows optical photographs (a, e, i, and m) of the four samples. The second and third columns display top‐view SEM images taken from selected central regions (a, e, i, and m) and edge regions (c, g, k, and o) as labeled in the optical images. The fourth column presents cross‐sectional SEM images (d, h, i, and p) of the four samples.

To further elucidate the effectiveness of the over‐lithiation strategy and analyze the failure mechanisms of AFLMBs, we examined the Li plating morphology on Cu current collectors in Cu||Li_1.0_NCM523 and Cu||Li_1.7_NCM523 cells after the 1st and 30th cycles (on fully charged state) using optical and SEM imaging. These samples are subsequently referenced as Li_1.0_NCM523‐Cu‐1st (**Figure** **6**a–d), Li_1.7_NCM523‐Cu‐1st (Figure 6e–h), Li_1.0_NCM523‐Cu‐30th (Figure 6i–l), and Li_1.7_NCM523‐Cu‐30th (Figure 6m–p), respectively.

The Li deposition of Li_1.0_NCM523‐Cu‐1st (Figure 6a–c) showed a pronounced heterogeneity across the Cu current collector. Some regions displayed a consistently granular Li morphology (Figure 6b, Region I), whereas other areas, with visible underlying Cu foil, exhibited significant Li dendritic growth (Figure 6c, Region II). This insufficient Li coverage on Cu foil, caused by the lack of active Li^+^ ions, led to the uneven electrode surface, negatively impacting Li ions diffusion. In stark contrast, the Li_1.7_NCM523‐Cu‐1st (Figure 6e) showed uniform Li deposition, with both the central (Figure 6f, Region III) and surrounding (Figure 6g, Region IV) areas displaying a homogeneously granular Li morphology. This demonstrates the substantial role of Li‐enriched cathodes in replenishing active Li^+^ ions storage in AFLMBs.

After 30 cycles, the morphological differences became even more pronounced. Li_1.0_NCM523‐Cu‐30th turned completely black (Figure 6i), leaving a large amount of dead lithium and a loose SEI layer (Figure 6j,k, Region V, VI), with its lithium layer reduced to a thickness of merely ≈2 µm (Figure 6l). This phenomenon resulted from unavoidable side reactions that consumed the limited active Li ions, impeding the migration of electrons and Li ions and accumulating dead Li. In contrast, Li_1.7_NCM523‐Cu‐30th remained a silver‐gray granular metallic lithium (Figure 6m–o, Region VII, VIII) that completely covered the Cu foil, with a Li deposition layer thickness of ≈25 µm (Figure 6p), indicating that a substantial amount of active Li remained after cycling.

Additionally, we compared the cycled cathodes of Li_1.0_NCM523‐30th and Li_1.7_NCM523‐30th. The Li_1.0_NCM523‐30th transitioned to a Li‐poor state, whereas Li_1.7_NCM523‐30th, due to its abundant Li storage, retained a structure and morphology identical to the original NCM523 (Figures S26–S30, Supporting Information).^[^ ^81^, ^82^, ^83^ ^]^ Clearly, the Li‐poor state of Li_1.0_NCM523‐30th results from the unavoidable Li loss during the cycling of AFLMBs, which further demonstrates the effectiveness of the Li‐abudant cathode strategy. The EIS also showed that Cu||Li_1.0_NCM523 exhibited a pronounced increase in impedance after only 10 cycles, while Cu||Li_1.7_NCM523 remained stable even after 50 cycles, benefiting from substantial Li reserves (Figure S31, Supporting Information). All the above results collectively demonstrated that the over‐lithiation strategy is a direct and effective way to extend the operational lifespan of AFLMBs significantly.

## 3 Conclusion

In summary, we systematically explored the over‐lithiation mechanism of Li_1+_ *_x_* NCM523 (0 ≤ *x* ≤ 3) cathode, unveiling its structural and elemental evolution behavior across a broad potential range of 4.3–0.5 V versus Li^+^/Li. It was found that moderate over‐lithiation facilitates the transition of lithium storage sites from octahedral to two tetrahedral, allowing the lattice to host up to twice the number of active Li^+^ ions. This process was accompanied by the sequential reductions of Ni^3+/2+^, Co^3+/2+^, and Mn^4+/2+^ to maintain charge balance. However, detailed morphological and structural analyses revealed that Li_1+_ *_x_* NCM523 might encounter particle fragmentation that correlates directly with the degree of lithiation. Below *x* ≤ 0.7, the particles remained dense and intact, but beyond *x* > 0.7, fragmentation occurred, increasing the contact area between the cathode and electrolyte, intensifying interfacial reactions, and degrading cycling stability. Our findings indicate that *x *= 0.7 represents the maximum tolerable lithiation threshold for Li_1+_ *_x_* NCM523. Further elemental valence analysis and DFT calculations revealed that the structural collapse and performance deterioration were caused by the excessive reduction of Mn^4+/2+^ (a key stabilizer for the layered structure), and even the metal displacement reactions involving Ni^2+/0^, Co^2+/0^, and Mn^2+/0^.

From this, we identified Li_1.7_NCM523 as the most optimal pre‐lithiated cathode, balancing additional lithium reserves to compensate for irreversible lithium losses and significantly enhancing the cycling life of AFLMBs. The Cu||Li_1.7_NCM523 cells demonstrated excellent capacity retention of 93.3% after 100 cycles at 0.5 C, and 78.5% after 200 cycles at 1 C, representing the best performance reported in the literature. However, it should be pointed out that the electrochemical prelithiation method involves cumbersome electrode rinsing and cell assembly/disassembly processes. As an innovative approach, chemical lithiation^[^ ^80^, ^84^, ^85^ ^]^ is more conducive to the scalable preparation of Li‐rich cathodes. Besides, electrolyte design^[^ ^86^, ^87^ ^]^ and current collector modification^[^ ^88^, ^89^ ^]^ are effective strategies for synergistically enhancing the electrochemical performance of AFLMBs. In future work, we will focus on providing a more streamlined method for preparing over‐lithiated cathodes, developing novel electrolytes and 3D current collectors to achieve zero volumetric strain, high specific energy, and long lifespan in AFLMBs.

## Conflict of Interest

The authors declare no conflict of interest.

## Supporting information



Supporting Information

## Data Availability

The data that support the findings of this study are available from the corresponding authors upon reasonable request
