# Supplementary material for: Unveiling the Over‐Lithiation Behavior of NCM523 Cathode Towards Long‐Life Anode‐Free Li Metal Batteries
Source: Adv Sci (Weinh). 2025 Mar 20;12(19):2503558. doi: 10.1002/advs.202503558 (PMC12097081; doi:10.1002/advs.202503558)
Supplement: Supplementary file 1 — Supporting Information [file ADVS-12-2503558-s001.docx]

**Supporting Information**

**Unveiling the Over-Lithiation Behavior of NCM523 Cathode Towards Long-life Anode-free Li Metal Batteries**

*Ruimin Gao^1#^, Minzhi Zhan^2#^, Tingcan Li^1^, Pei Xiong^3^, Qian Zhang^1^, Zhefeng Chen^2^, Jike Wang^3^, Xinping Ai^1^, Feng Pan^2^*, Liumin Suo^4, 5^*, Jiangfeng Qian^1^**

*^1^ Hubei Key Laboratory of Electrochemical Power Sources, College of Chemistry and*

*Molecular Sciences, Wuhan University, Wuhan, Hubei 430072, China;*

*^2^ School of Advanced Materials, Peking University, Shenzhen Graduate School, Shenzhen 518055, China;*

*^3^ The Institute for Advanced Studies, Wuhan University, Wuhan, Hubei, 430072, China.*

*^4^ Institute of Physics, Chinese Academy of Sciences, Beijing National Laboratory for Condensed Matter Physics, Beijing 100190, China*

*^5^ Center of Materials Science and Optoelectronics Engineering, University of Chinese Academy of Sciences, Beijing 100049, China.*

# These authors contribute equally to this work.

*Corresponding authors. E-mail: panfeng@pkusz.edu.cn (F. Pan); suoliumin@iphy.ac.cn (L. M. Suo); [jfqian@whu.edu.cn](mailto:jfqian@whu.edu.cn) (J. F. Qian)

**Keywords:** Anode-free Li metal batteries, Li-enriched NCM523 cathode, Over-lithiation behavior, Structure evolution, Valence state evolution

**Experimental section**

**Materials:**

Commercial Li_1.0_Ni_0.5_Co_0.2_Mn_0.3_O_2_ (NCM523) powders were procured from Guizhou Zhenhua E-Chem Co., Ltd. Li metal chips (1 mm thick, 15.6 mm in diameter) were obtained from Guangdong Canrd New Energy Technology Co., Ltd. Commercial Cu foil (10 μm) was purchased from Colleague Hardware Co., Ltd. The electrolyte solution, of battery-grade quality, was supplied by Suzhou Duoduo Chemicals Co., Ltd. Over-lithiated Li_1.7_NCM523, Li_2.0_NCM523, Li_3.85_NCM523 was synthesized using an electrochemical method. Li||Li_1.0_NCM523 half-cell were over-discharged to 1.2 V, 0.9 V, and 0.5 V, respectively, followed by disassembled within a glove box to retrieve the desired Li_1+_*_x_*NCM523 cathodes. These cathodes were meticulously cleaned with anhydrous DME solvent (2 mL) to eliminate any residual electrolytes and salts. Subsequently, they were subjected to vacuum drying before being sealed in airtight containers within the glovebox. All reagents and solvents were provided by Aladdin Company and used without further purification.

A convenient and practical chemical lithiation method was employed to prepare Li_1.7_NCM523 (FL-Li). Initially, a 0.1 M 9-fluorenone lithium/tetrahydrofuran solution (FL-Li/THF) was prepared by dissolving FL powder and Li metal with a molar ratio of 1:2 in tetrahydrofuran solvent. Subsequently, the Li_1.0_NCM523 electrode sheets were immersed in an appropriate amount of the chemical lithiation reagent and allowed to react thoroughly for 10 min. After the reaction, the Li_1.7_NCM523 (FL-Li) were rinsed three times with anhydrous THF and dried for further use.

**Morphological and Structural Characterization:**

X-ray diffraction (XRD) analysis was performed on Li_1+_*_x_*NCM523 using the Rigaku Smart Lab SE instrument. Cu Kα radiation was utilized with a scanning speed of 5° min^-1^, ranging from 10° to 80°. The morphologies of Li_1+_*_x_*NCM523 the were examined using scanning electron microscope (SEM, Zeiss Merlin Compact). For Transmission Electron Microscopy (TEM, JEM-NEOARM) test, all Li_1+_*_x_*NCM523 samples were prepared by using a focusing ion beam (FIB, TESCAN SOLARIS) with sample thicknesses being less than 100 nm for TEM work appropriateness. X-ray photoelectron spectroscopy (XPS) (ESCALAB Xi+, Thermo Fisher Scientific) was employed to analyze the valence state of Li_1+_*_x_*NCM523, utilizing monochromatic Al Kα radiation (225 W, 15 mA, 15 kV), Ar^+^ etching was performed for 180 s to completely eliminate interference from the surface layer. Time-of-flight secondary-ion mass spectrometry (TOF-SIMS, Tescan AMBER) was measured to reveal the uniform pre-lithiation of Li_1+_*_x_*NCM523.

**X-ray Absorbtion Spectra (XAS) Characterization:**

Transmission hard X-ray absorption spectroscopy (XAS) was performed using a laboratory easyXAFS300+ device (easyXAFS LLC) to determine the material bulk electronic structure. Absorption spectra at the Co K-edge, Ni K-edge, and Mn K-edge were directly acquired from electrode samples and compared with standard foil and reference samples of known oxidation states. To ensure the reliability of the results, each measurement was repeated at least twice to confirm reproducibility. The X-ray absorption near edge structure (XANES) data underwent background subtraction and normalization using the AUTOBK routine within the Athena software. Detailed insights into the spatial distribution of neighbouring atoms surrounding the transition metal (TM) atoms were extracted from the extended absorption fine structure (EXAFS) data. The EXAFS χ-functions were then isolated from the raw experimental data through a well-established data reduction method implemented in the IFEFFIT software.

**Electrochemical Measurements:**

To fabricate the cathode electrode, Li_1.0_NCM523 (80wt%), super P (10wt%), and polyvinylidene fluoride (PVDF, 10wt%) were mixed in N-methyl-2-pyrrolidone (NMP). The obtained slurry was cast onto the carbon-coated aluminum foil and then dried at 80 ℃ in a vacuum oven for over 12 h. The mass loading of active materials in the electrodes film was ≈ 10 mg cm^-2^ and the cathodes were assessed by coin-type (CR2032) cells with metal Li in half-cells or Cu foil in AFLMBs as anodes, Celgard 2400 Polyethylene (PE) membrane as the separator. The 1.0 M LiPF_6_ dissolved in a 1:1:1 volume ratio of ethylene carbonate: ethyl methyl carbonate: dimethyl carbonate with a 5% vinylene carbonate additive was the electrolyte for Li||Li_1+_*_x_*NCM523 half-cells. An ether-based high-concentrated electrolyte with a molar ratio of bis(fluorosulfonyl)imide lithium (LiFSI), lithium nitrate (LiNO_3_), and dimethoxyethane (DME) of 9:1:20 was used in Cu||Li_1+_*_x_*NCM523 AFLMBs. A non-flammable electrolyte, which composition is 1.27 M of LiFSI dissolved in TEP, DME and TTE, with the ratio as 21:4:75 v:v:v % (LHCE-TEP-Et) was used in Cu||Li_1+_*_x_*NCM523 AFLMBs to further meet the demands for enhanced safety and practical applications. Electrochemical cycling measurements and galvanostatic intermittent titration technique (GITT) were performed on a LAND-CT 3001A multichannel battery tester at 25 °C. The electrochemical impedance spectroscopy (EIS) was carried out on Admiral electrochemical workstation (Metrohm Co., Ltd.), covering a frequency range of 10^-2^ to 10^5^ Hz with an amplitude of 10 mV.

**Computational Details:**

DFT calculations are implemented in the Vienna ab initio simulation package (VASP) with the projector augmented wave (PAW) method^[1, 2, 3]^. The exchange and correlation energy of electrons is described using the generalized gradient approximation (GGA) with the functional of Perdew-Burke-Ernzerhof (PBE) formulation^[4, 5]^. To account for the strong on-site Coulomb interaction of the TMs 3d electrons, PBE+U approach is used to calculate structural and electronic properties with spin polarization^[6, 7]^. The U values of Ni, Co and Mn are set at 6.2, 3.3, 3.9 eV according to previous studies and Materials Project^[8, 9]^ and the magnetic orders of TMs are set to be ferromagnetic. A 520 eV plane-wave energy cut-off is used and Monkhorst-Pack k-point grids of at least 1000/Natom in the Brillouin zone are set for all calculations. The convergence criteria for the force and energy are set to 0.02 eV/Å and 1 ×10^-5^ eV per atom. The crystal orbital Hamilton population (COHP)^[10]^ analyses are performed by Local Orbital Basis Suite Towards Electronic-Structure Reconstruction (LOBSTER) code^[11]^.

Li_32_Ni_17_Co_6_Mn_9_O_64_ and Li_64_Ni_17_Co_6_Mn_9_O_64_ supercells are built for structure relaxation and electronic properties calculation. The stoichiometry slightly differs from that in actual material because the number of elements in an individual crystal cell must be an integer during the calculations. The TM cations arrangement pattern in supercell models, maintain consistency between Li_1.0_NCM523 and Li_2.0_NCM523, is generated by special quasi-random structure (SQS) method.


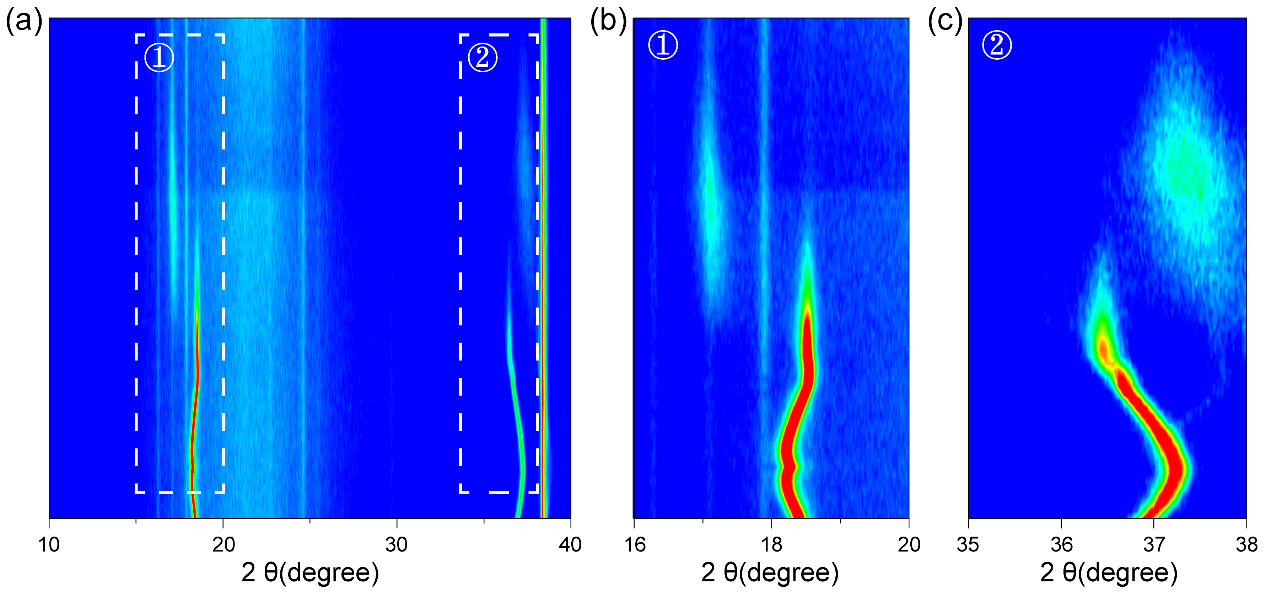


**Figure S1:** Contour plot of in-situ XRD patterns of an Li||Li_1.0_NCM523 half-cell within the 2 θ degree of a) 10-40°; b) 16-20°; c) 35-38°.

In the *in-situ* XRD measurement, the Li||Li_1.0_NCM523 half-cell is charged to 4.3 V and then discharge to 0.5 V at a current density of 0.5 C, using a commercial carbonate ester electrolyte (1 M LiPF_6_ in EC/DEC/EMC). This *in-situ* XRD clearly illustrate the structural evolution of Li_1.0_NCM523 during the over-discharge process, which transform from Li1 phase → the coexistence of Li1 and Li2 phases → Li2 phase.


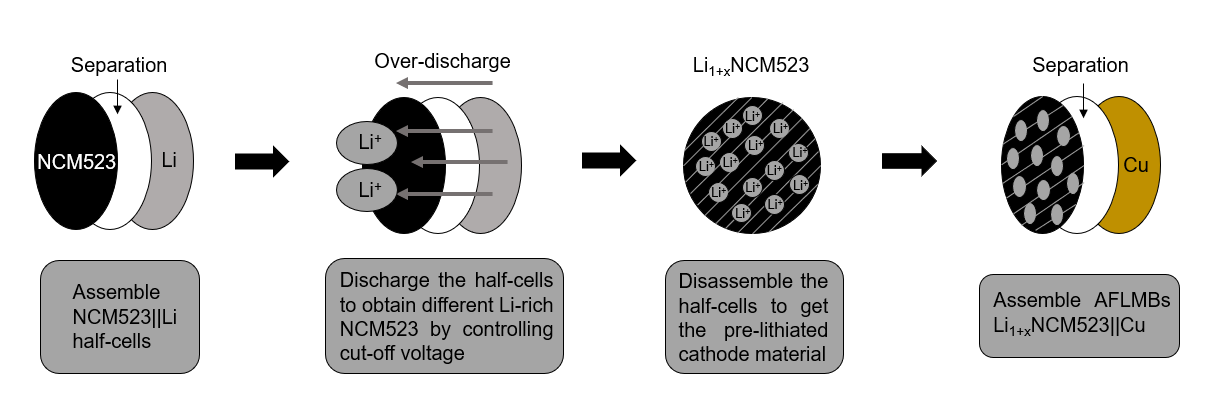


**Figure S2**：The schematic diagram of the electrochemical pre-lithiation process.


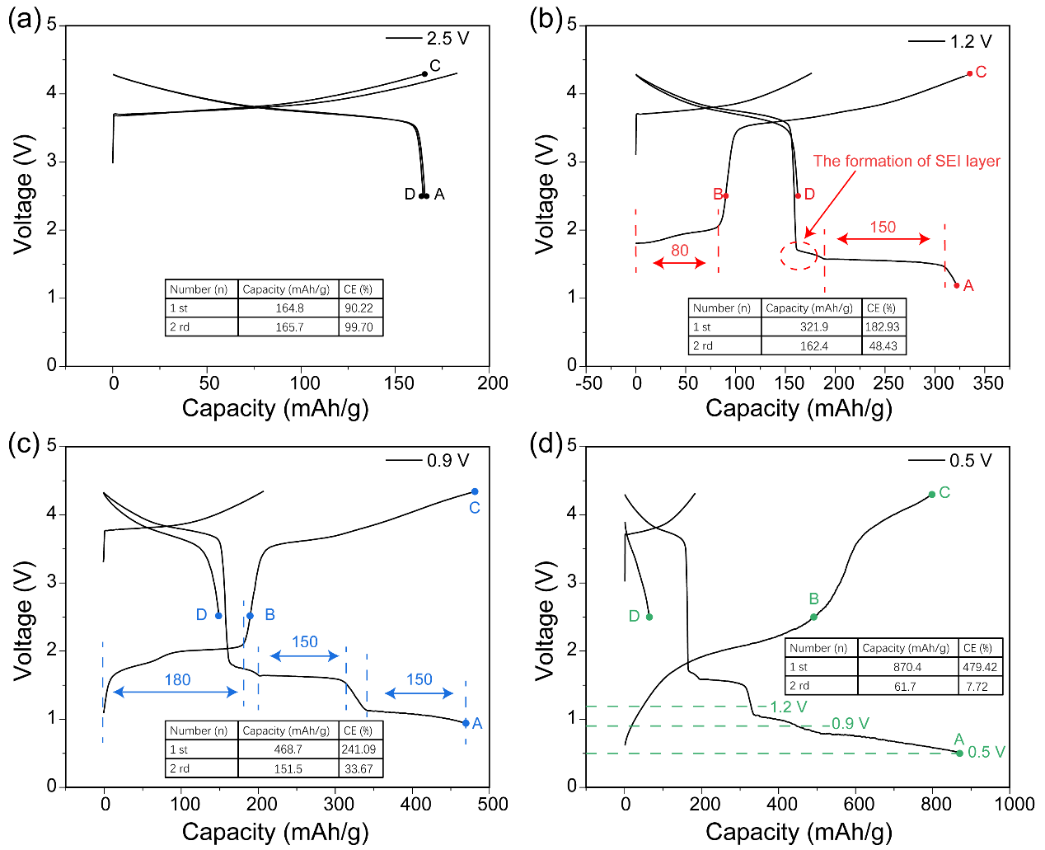


**Figure S3**：The 1^st^ and 2^nd^ charge/discharge curves of a) NCM523-2.5 V (Li_1.0_NCM523); b) NCM523-1.2 V (Li_1.7_NCM523); c) NCM523-0.9 V (Li_2.0_NCM523) and d) NCM523-0.5 V (Li_3.85_NCM523).

In Figure S3, the charge/discharge curve of Li_1.0_NCM523 appear as a smooth slope, showing a first discharge capacity of 164.8 mAh g^-1^ (**Figure S3a**). Following pre-lithiation, Li_1.7_NCM523 has a new discharge plateau at 1.5 V, which significantly boosted its initial discharge capacity to 318.8 mAh g^-1^. During the charging process, a portion of the additional Li^+^ ions is released, showing a corresponding charging plateau at 1.8 V with a capacity of ~80 mAh g^-1^. Subsequently, the remaining Li^+^ ions are fully released at higher voltages (above 4.0 V), resulting in a notable increase in charge capacity to 335 mAh g^-1^ (**Figure S3b**).

In comparison to Li_1.7_NCM523, Li_2.0_NCM523 exhibit another additional discharge plateau at 1.0 V, with the initial discharge capacity further increasing to 468.7 mAh g^-1^. A similar phenomenon is observed during charging. The Li^+^ ions partially de-intercalate at low voltage (1.8 V and 2.0 V), providing partial charge capacity of 180 mAh g^-1^, and fully de-intercalate at higher voltage (above 4.0 V), reaching 345 mAh g^-1^, which is almost equal to its discharge capacity (**Figure S3c**). Obviously, these electrochemical behaviors offer compelling evidence for the reversibility of the pre-lithiation process.

Upon further discharge to 0.5 V, Li_3.85_NCM523 exhibit abnormal charge/discharge curve and exceptionally high irreversible capacity (**Figure S3d**). This observation indicate that over-discharging can potentially cause irreversible decomposition of the cathode material.


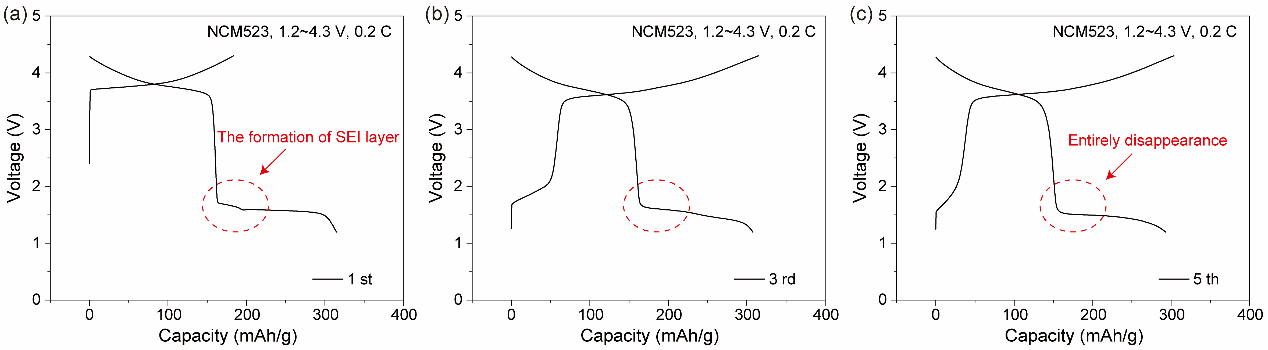


**Figure** **S4**: The a) 1^st^; b) 2^rd^; and c) 5^th^ charge/discharge curves of Li_1.0_NCM523 in the voltage range of 1.2-4.3 V at 0.2 C. The plateau at 1.65 V gradually diminish after several cycles, indicating it corresponding to the formation of SEI layer.


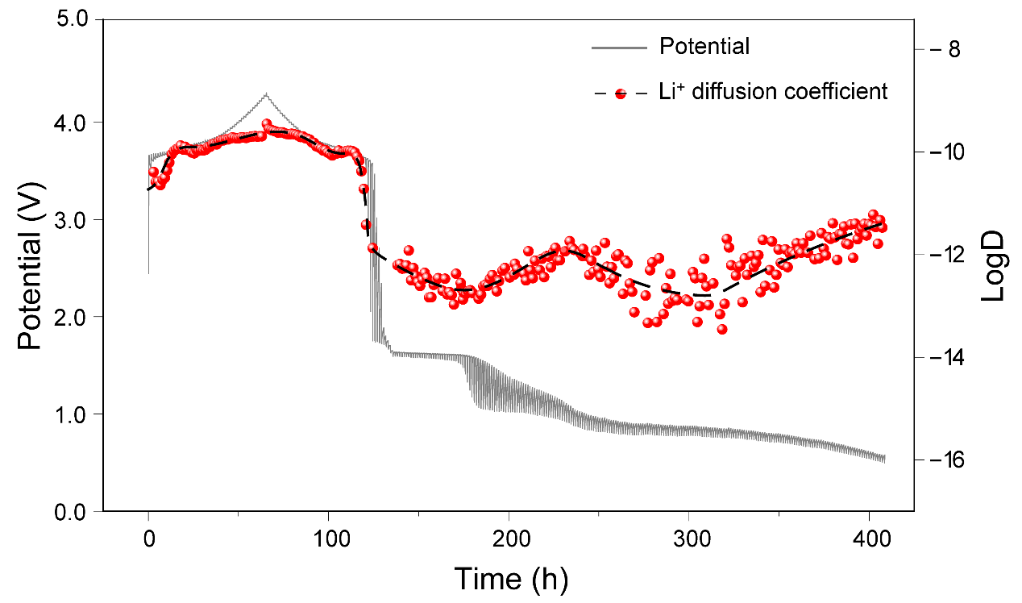


**Figure** **S5**：The Galvanostatic Intermittent Titration test (GITT) of Li_1.0_NCM523 at 0.1 C in the voltage range of 4.3-0.5 V.

It is clear that the Li^+^ ions diffusion coefficient within the over-discharge voltage range (2.5 V-0.5 V, 10^-12^) is significantly lower than that within the standard operational voltage window (4.3 V-2.5 V, 10^-10^). This reduction is primarily caused by the pre-lithiation process, which gradually fill available lithium vacancies, thereby increasing the electrostatic repulsion among the Li^+^ ions. As a result, the heightened electrostatic forces raise the energy barrier for Li^+^ ions migration. During deep discharge (below 0.9 V), the Li^+^ ions diffusion coefficient exhibits an anomalous increase, likely due to the fragmentation of cathode particle, which has reduced volume (shortening the Li^+^ ions diffusion path) and increased specific surface area (providing additional migration pathways for Li^+^ ions).


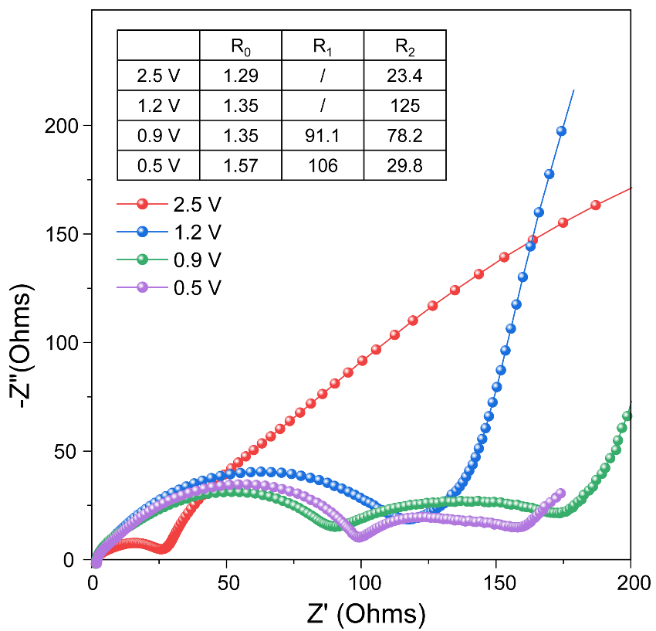


**Figure** **S6**：The Electrochemical Impedance Spectroscopy (EIS) of Li_1.0_NCM523, Li_1.7_NCM523, Li_3.85_NCM523 and Li_2.0_NCM523.

The Electrochemical Impedance Spectroscopy (EIS) results demonstrate that the impedance of all Li_1+_*_x_*NCM523 samples are consistently higher than that of the original Li_1.0_NCM523. The order is R_(Li1.0NCM523)_ < R_(Li1.7NCM523)_ < R_(Li3.85NCM523)_ < R_(Li2.0NCM523)_. This increase in impedance can be attributed to structural changes induced by the pre-lithiation. Both Li_1.0_NCM523 and Li_1.7_NCM523 exhibit single-semicircle Nyquist plots, which are indicative of charge transfer impedance. However, following over-discharge, the SEI film on the surface of the Li_2.0_NCM523 and Li_3.85_NCM523 thicken considerably, result in two-semicircle Nyquist plots, corresponding to the Li^+^ ions diffusion/migration impedance within the SEI layer and the charge transfer impedance, respectively. In addition, Li_3.85_NCM523 undergo severe pulverization due to irreversible structural damage. This pulverization increases its specific surface area while reducing its particle volume, leading to the abnormal order that R_(Li3.85NCM523)_ < R_(Li2.0NCM523)_.


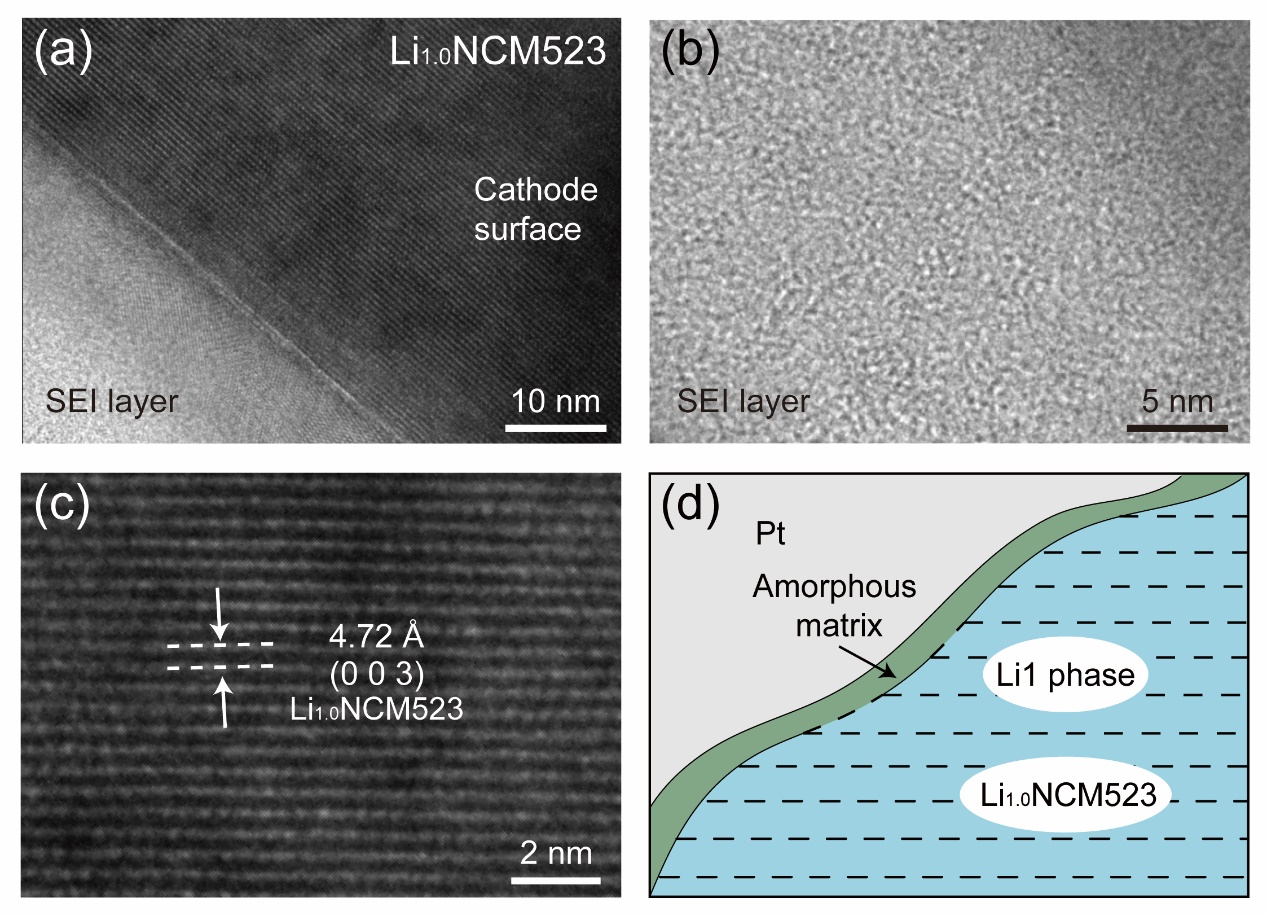


**Figure** **S7**: The TEM analyses of Li_1.0_NCM523, which includes: a) the interface between the Li_1.0_NCM523 and the SEI layer; b) the SEI layer; c) the internal structure and d) a schematic diagram illustrating the morphology of Li_1.0_NCM523.

The TEM images reveal that the SEI layer of Li_1.0_NCM523 is entirely composed of amorphous organic phase (**Figure S7b**), while the crystal structure of Li_1.0_NCM523 is characterized by a single Li1 phase with a lattice spacing of 4.73 Å, corresponding to the (003) characteristic peak (**Figure S7c**).


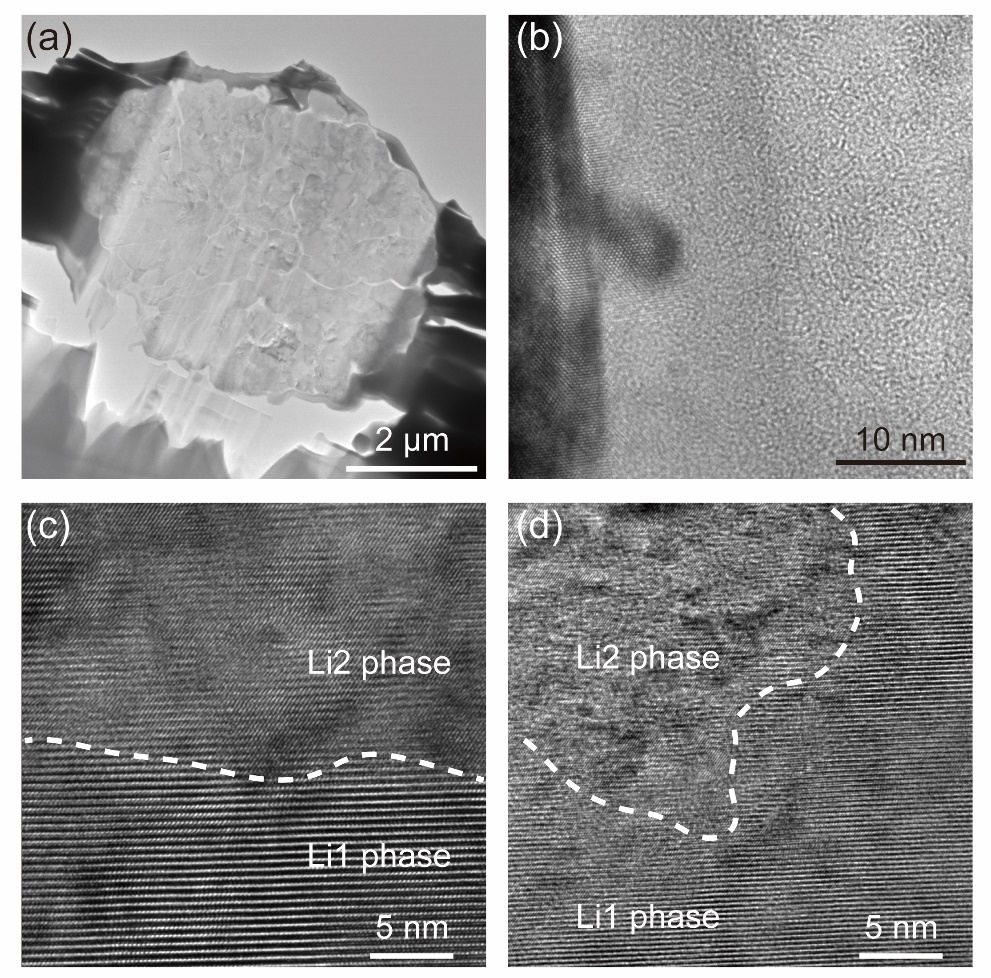


**Figure** **S8**: The TEM analyses of Li_1.7_NCM523, which includes: a) a cross-sectional view obtained via focused ion beam (FIB) slicing; b) the SEI layer of Li_1.7_NCM523; c-d) the internal structure of Li_1.7_NCM523 at a large scale.


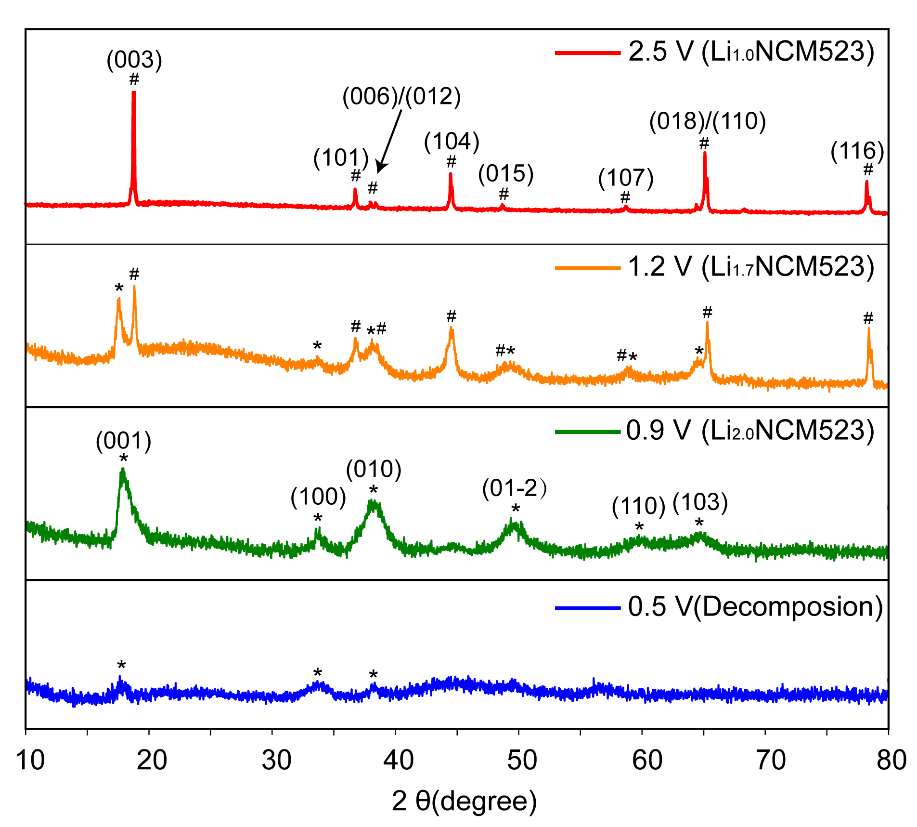


**Figure** **S9**: The X-ray powder diffraction (XRD) of Li_1+_*_x_*NCM523.

The XRD show that Li_1.0_NCM523 exhibit typical Li1 phase (isostructural with α-NaFeO_2_) characteristic peaks, including (003), (101), (006)/(012), (104), etc. The coexistence of Li1 and Li2 phases is observed in Li_1.7_NCM523, demonstrating its biphasic structure. Li_2.0_NCM523 show a single Li2 phase (isostructural with Li_2_NiO_2_) with characteristic peaks at (001), (100), and (101), etc. Li_3.85_NCM523 almost completely transforms into an amorphous phase due to excessive pre-lithiation, which irreversibly damage its crystal structure.


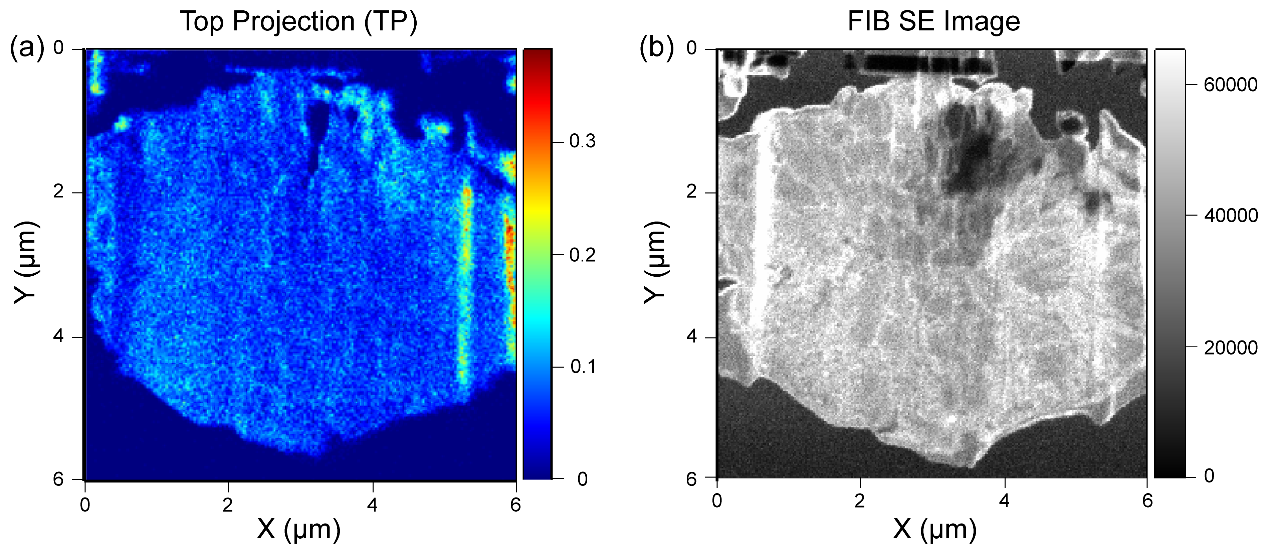


**Figure** **S10**: Time-of-flight secondary ion mass spectrometry (TOF-SIMS) analysis of Li_1.7_NCM523 after over-discharge to 1.2 V.

TOF-SIMS analysis of Li_1.7_NCM523 indicate that additional Li^+^ ions are uniformly distributed throughout the entire cross-section, with no lithiation gradient is observed between the material surface and its interior, demonstrating that the pre-lithiation is uniform and complete.


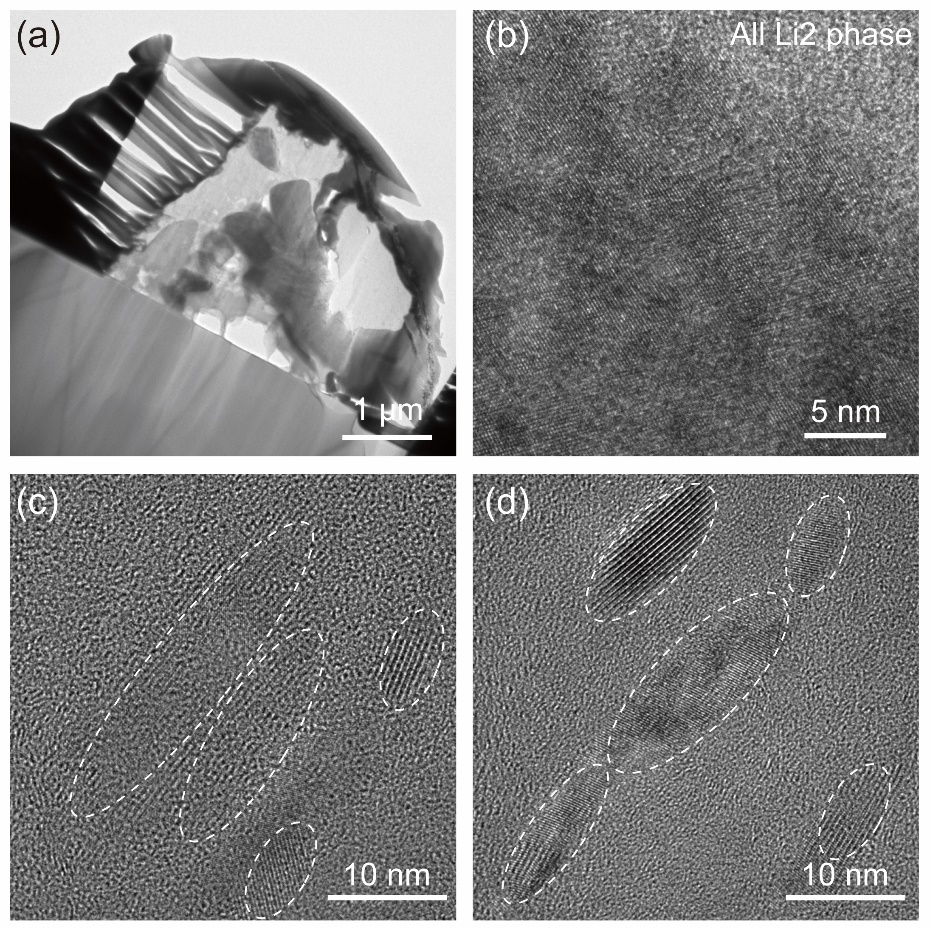


**Figure** **S11**：The TEM analyses of Li_2.0_NCM523, which includes: a) a cross-sectional view obtained via focused ion beam (FIB) slicing; b) the internal structure of Li_2.0_NCM523; c-d) the SEI layer of Li_2.0_NCM523.


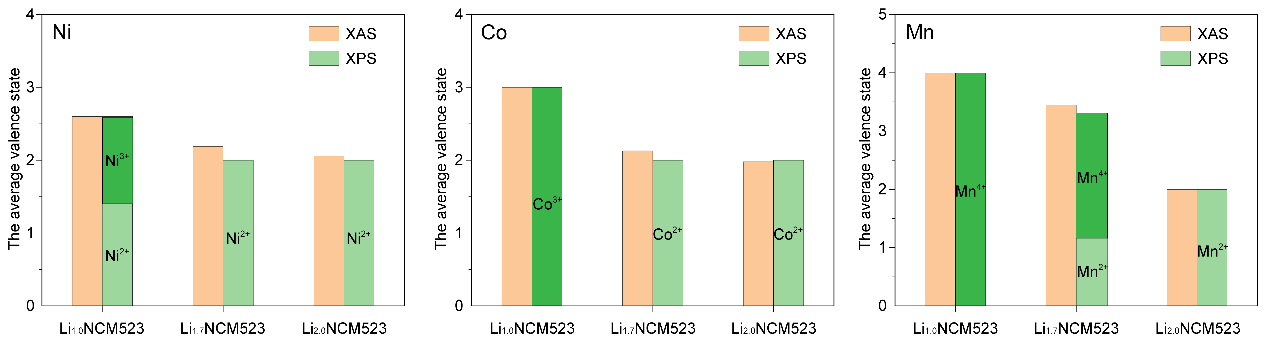


**Figure S12**: A comparison between the average valence state of TM elements derived from XAS and XPS measurements.

XAS has a larger penetration depth, making it suitable for analyzing bulk materials and studying the internal electronic structure and coordination environment. In contrast, XPS has a shallower penetration depth (approximately 1-10 nm), making it more appropriate for determining the chemical composition, valence states, and electronic states of surface elements. However, XPS offers higher energy resolution (up to 0.1 eV), enabling precise differentiation of subtle chemical state differences. Therefore, XAS and XPS data exhibit strong complementarity. Combining these two characterization methods for the analysis of Li_1+x_NCM523 facilitates a comprehensive and in-depth understanding of the reduction trends of TM (transition metal) elements and the evolution patterns of their average valence states from multiple perspectives. The high consistency observed between XPS and XAS data further reinforces the credibility of the conclusions.


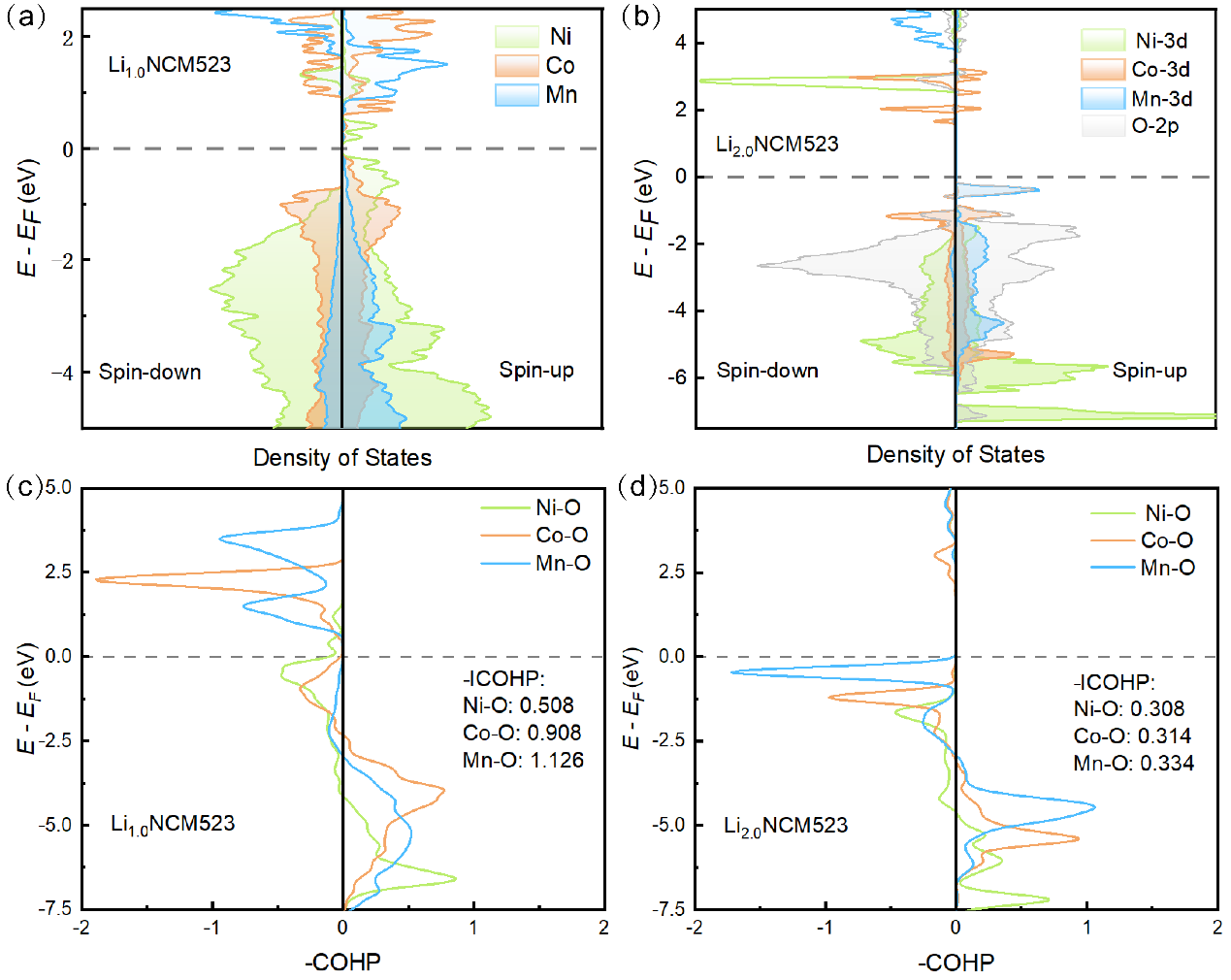


**Figure** **S13**：Partial density of states (pDOS) of TM-3d and O-2p in (a) Li_1.0_NCM523 and (b) Li_2.0_NCM523; Crystal orbital Hamilton population (COHP) of TM-O bonds in (c) Li_1.0_NCM523 and (d) Li_2.0_NCM523.


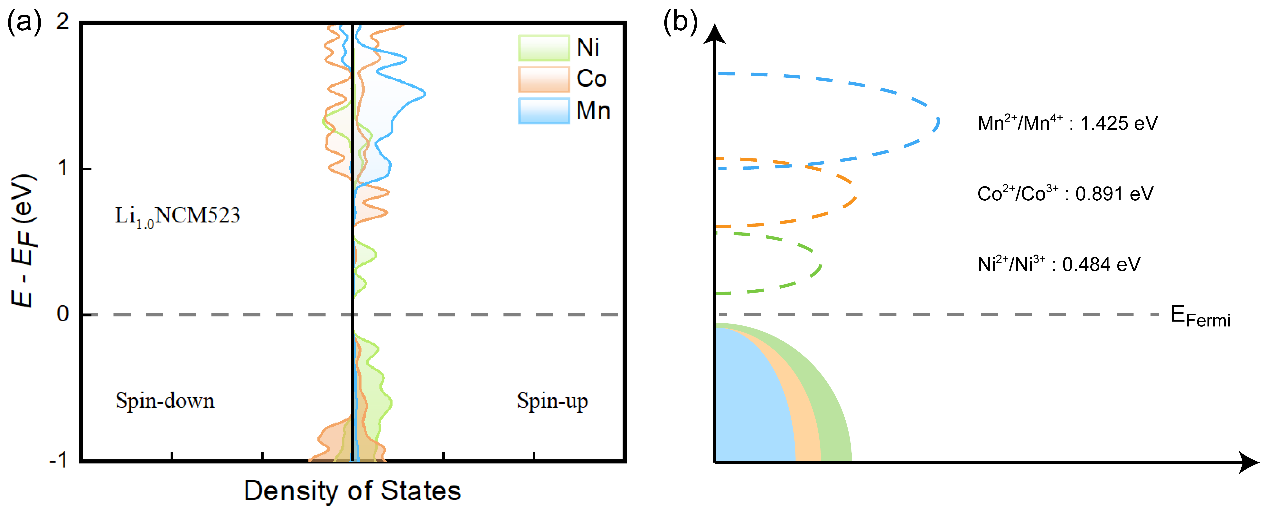


**Figure** **S14**: (a) pDOS of TM cations near fermi level; (b) Scheme of pDOS of TM cations near fermi level.

The COHP calculation and pDOS show that the unoccupied states of the TM-d bands are hybridized with the O-2p bands. The states near the Fermi level are identified as antibonding states, which correspond to the oxidation-reduction couples of TM cations. As the Fermi level shift during pre-lithiation, the TM-O bonds weaken. The average energy of the TM-O antibonding states near the Fermi level is calculated to confirm the reduction order of the TM cations (Ni＞Co＞Mn).


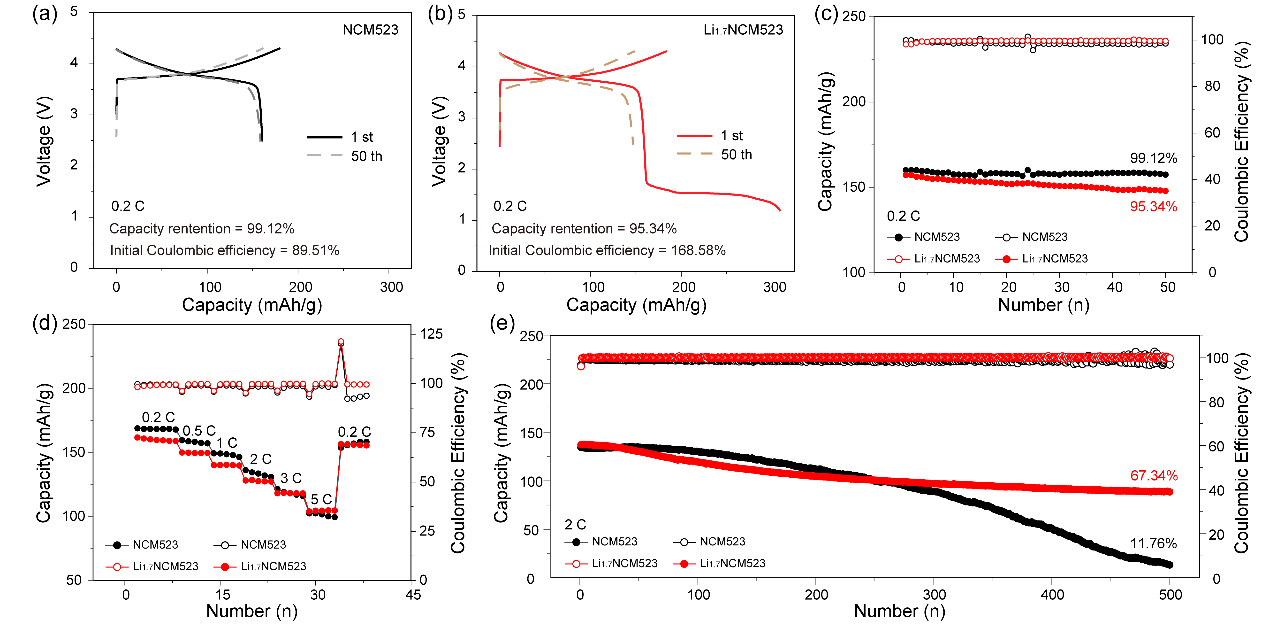


**Figure** **S15**: The half-cell electrochemical performances of Li||Li_1.0_NCM523 and Li||Li_1.7_NCM523. The charge/discharge curve of (a) Li||Li_1.0_NCM523 and (b) Li||Li_1.7_NCM523 at 0.2 C; (c) The cycling performance at 0.2 C and (d) rate performance and (e) long-cycling stability of Li||Li_1.0_NCM523 and Li||Li_1.7_NCM523 at 2 C. All tests are conducted at ambient temperature (25 °C) using a commercial ester electrolyte within a voltage range of 2.5-4.3 V.

To evaluate the intrinsic electrochemical properties of Li_1.7_NCM523, the half-cells utilizing Li metal anode is assembled. The cycling performance of Li||Li_1.7_NCM523 (95.34%) is very close to that of Li||Li_1.0_NCM523 (99.12%) at 0.2 C after 50 cycles (**Figure S14a-c**). The rate performance (**Figure S14d**) indicates that the electrochemical performance of Li||Li_1.7_NCM523 is slightly inferior to Li||Li_1.0_NCM523 at low test rates (0.2 C-2 C). However, at higher test rates (3 C-5 C), Li||Li_1.7_NCM523 outperform Li||Li_1.0_NCM523. At a high rate of 2 C over 500 cycles (**Figure S14e**), Li||Li_1.7_NCM523 has a capacity retention of 67.34%, significantly surpassing Li_1.0_NCM523||Li, which exhibit a rapid capacity decline to 11.76% after 200 cycles. Consequently, the pre-lithiation process does not compromise the cycling stability of Li_1.7_NCM523.


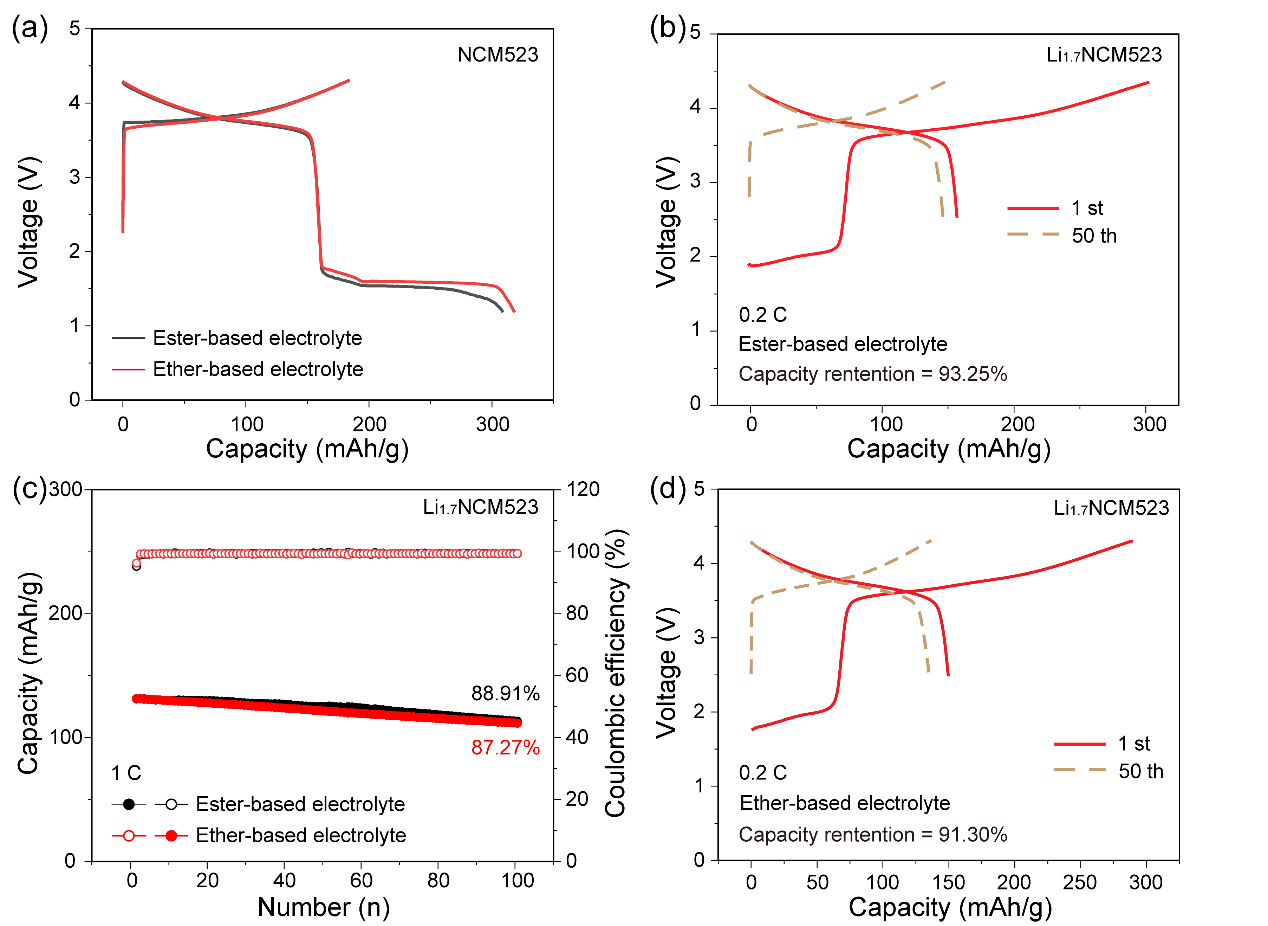


**Figure S16:** The half-cell electrochemical performances of Li||Li_1.7_NCM523 with different electrolytes. (a) Over-discharge curve of Li||Li_1.7_NCM523 in 4.3~1.2 V at 0.2 C. The charge/discharge curve of Li||Li_1.7_NCM523 with (b) the commercial ester-based electrolyte and (d) high-concentrated-ether-based electrolytes. (c) The comparison of the cycling stability of Li||Li_1.7_NCM523 using the two electrolytes mentioned above.

As shown in Figure S15a, NCM523 exhibited very similar over-discharge behavior when using ether-based and ester-based electrolytes. The cycling performance of Li||Li_1.7_NCM523 in both mentioned above electrolytes is also nearly identical, as shown in Figure S15b-d. This suggests that the type of electrolyte is not the primary factor influencing the half-cell performance.


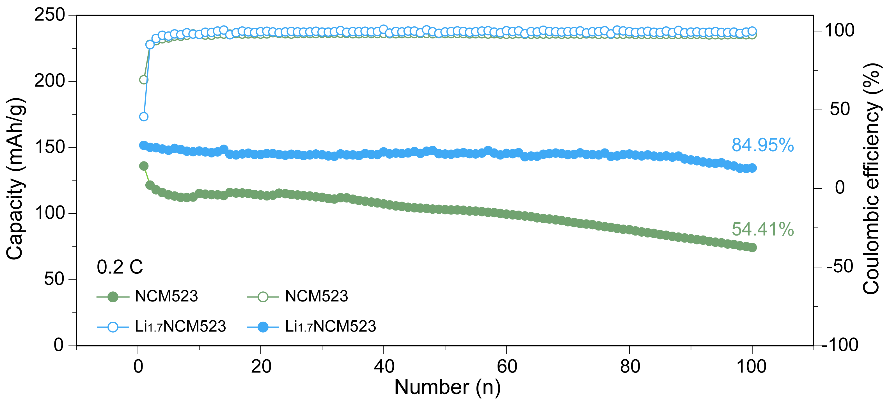


**Figure** **S17**: The cycling stability of Cu||Li_1.0_NCM523 and Cu||Li_1.7_NCM523 at 0.2 C.

All AFLMBs utilize an ether-based high-concentrated electrolyte, owing to its excellent Li compatibility and high coulombic efficiency. The composition of this electrolyte comprises a molar ratio of bis(fluorosulfonyl)imide lithium (LiFSI), lithium nitrate (LiNO_3_), and dimethoxyethane (DME) at 9:1:20, respectively. All tests are conducted within a voltage range of 2.5-4.3 V and at a constant temperature of 25 °C.


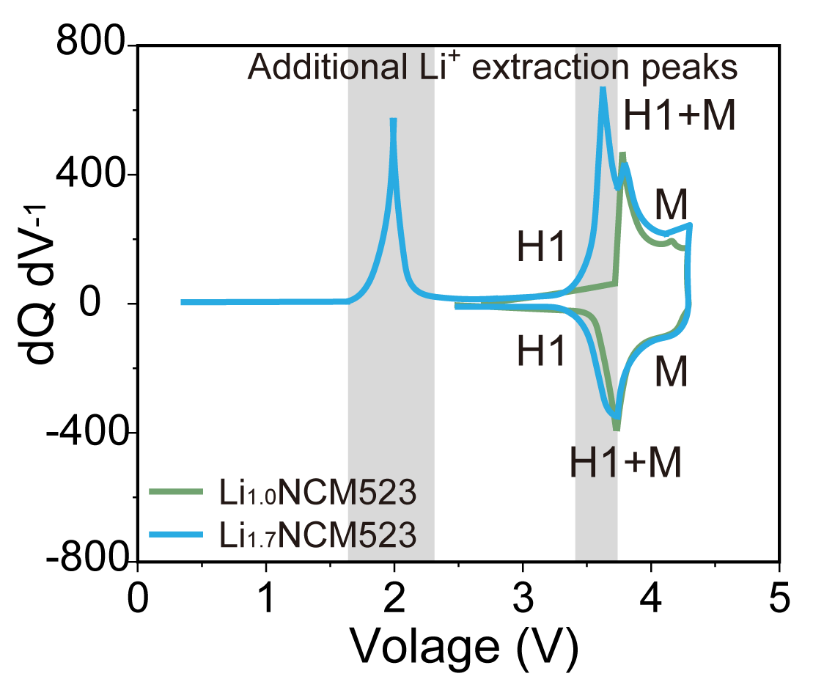


**Figure** **S18**: The dQ/dV curves of Cu||Li_1.0_NCM523 and Cu||Li_1.7_NCM523.


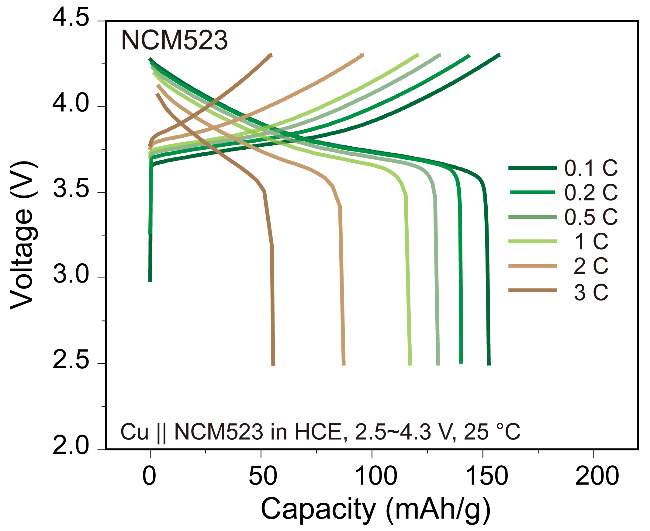


**Figure** **S19**: The rate performance of Cu||Li_1.0_NCM523, which exhibit poor rate performance, particularly at high test rate.


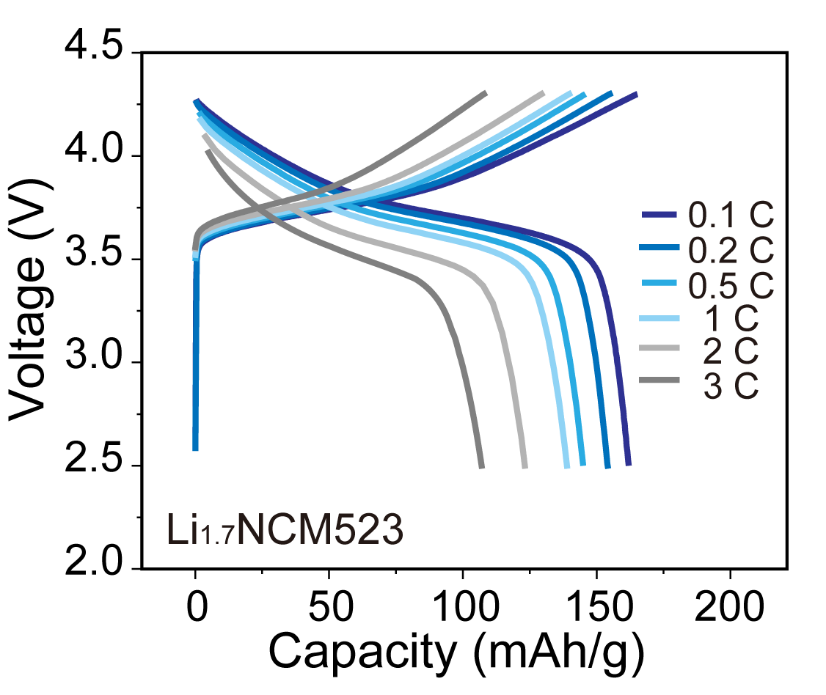


**Figure** **S20**: The rate performance of Cu||Li_1.7_NCM523 in 2.5-4.3 V.


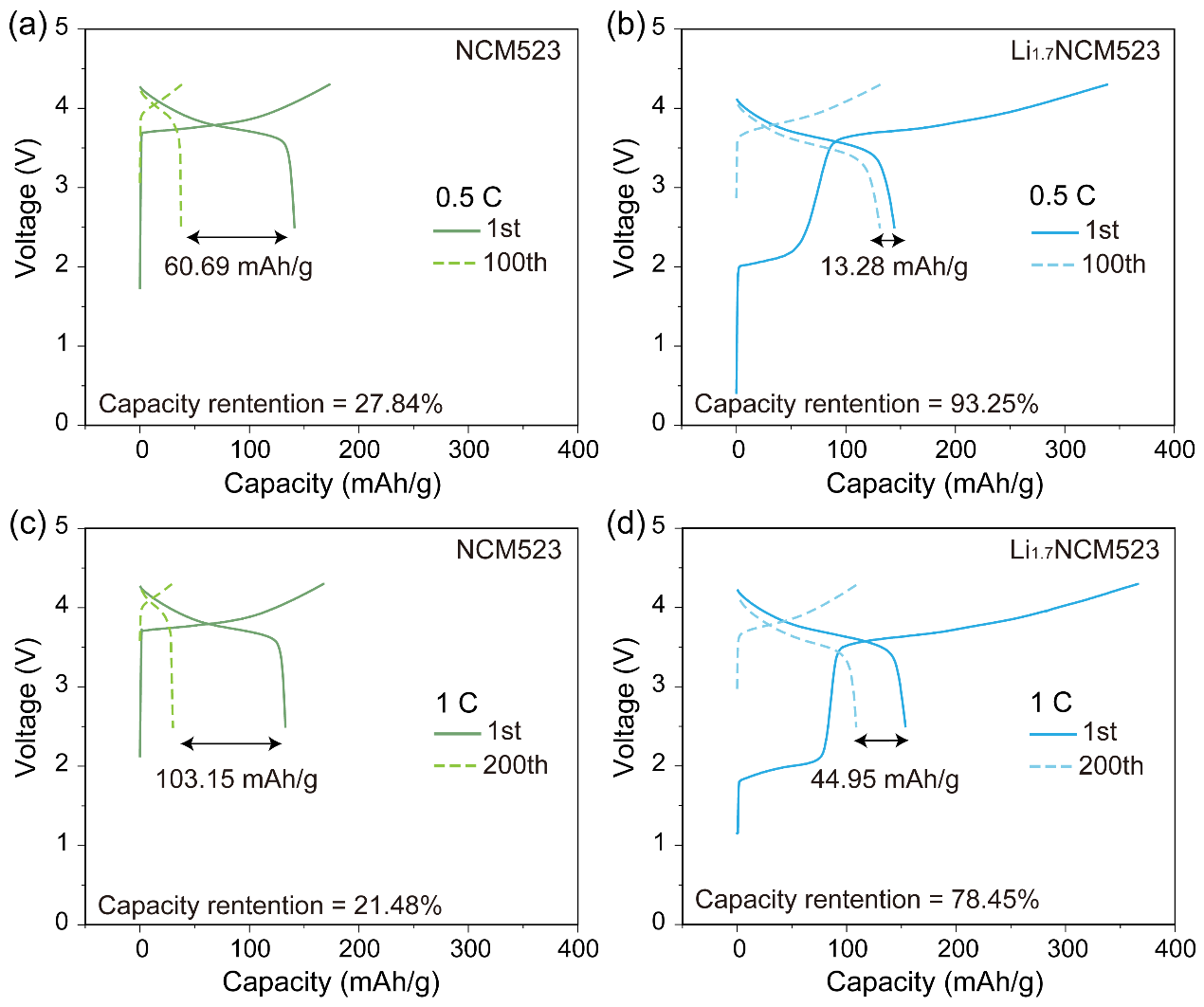


**Figure** **S2****1**: The charge/discharge curves of Cu||Li_1.0_NCM523 at (a) 0.5 C and (c) 1 C. The charge/discharge curves of Cu||Li_1.7_NCM523 at (b) 0.5 C and (d) 1 C (solid line: 1^st^ cycle; dashed line: 200^th^ cycle). Before long-term cycling, all AFLMBs are activated at a low rate of 0.2 C for three cycles.


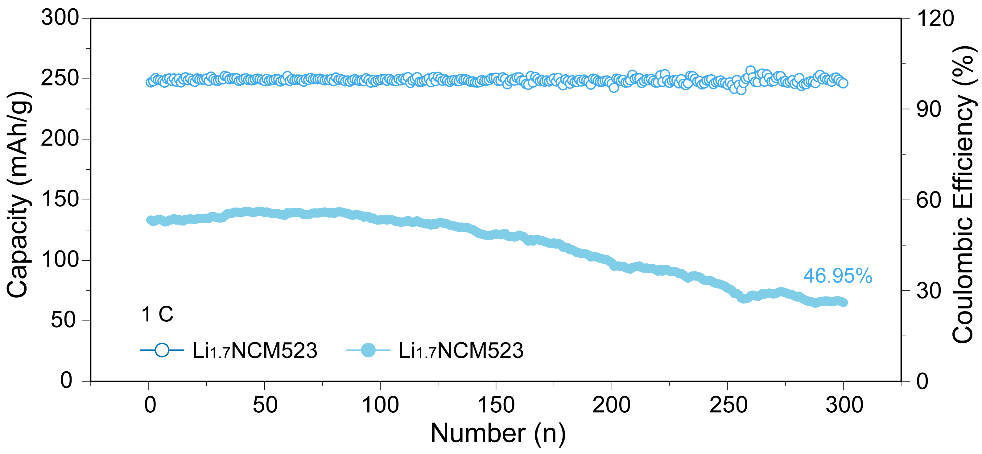


**Figure** **S22**: The cycling stability of Cu||Li_1.7_NCM523 at 1 C.

After 100 cycles, Cu||Li_1.7_NCM523 maintain an excellent capacity retention of 95.33%. However, this retention decreases to 78.45% after 200 cycles and further declines to 46.95% after 300 cycles. The decline in capacity retention is attributed to the depletion of additional Li^+^ ions during long-term cycling.


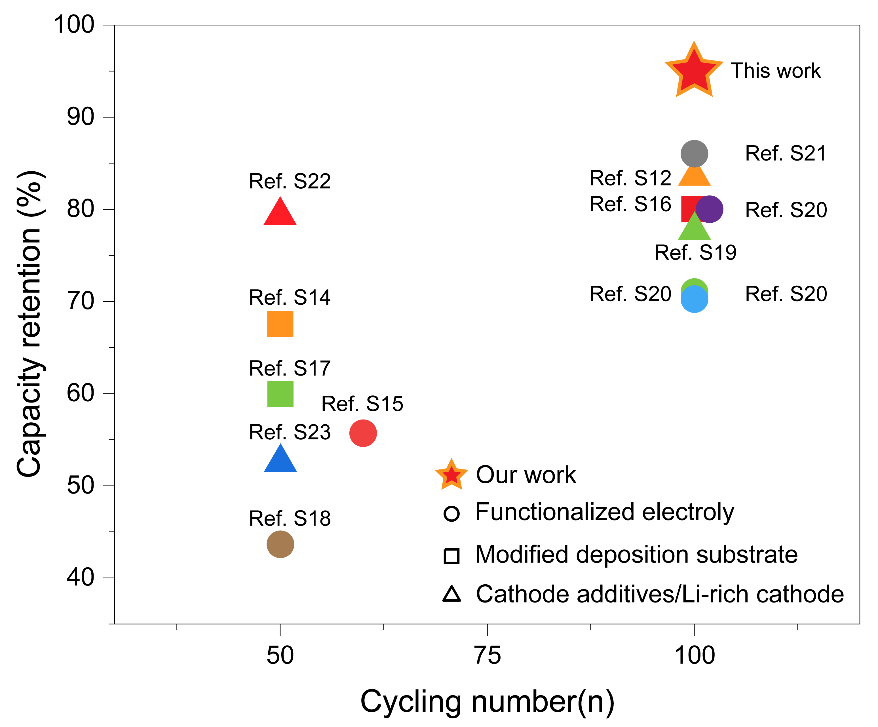


**Figure** **S23**：Comparison of our Cu||Li_1.7_NCM523 AFLMBs with other reported cell systems.


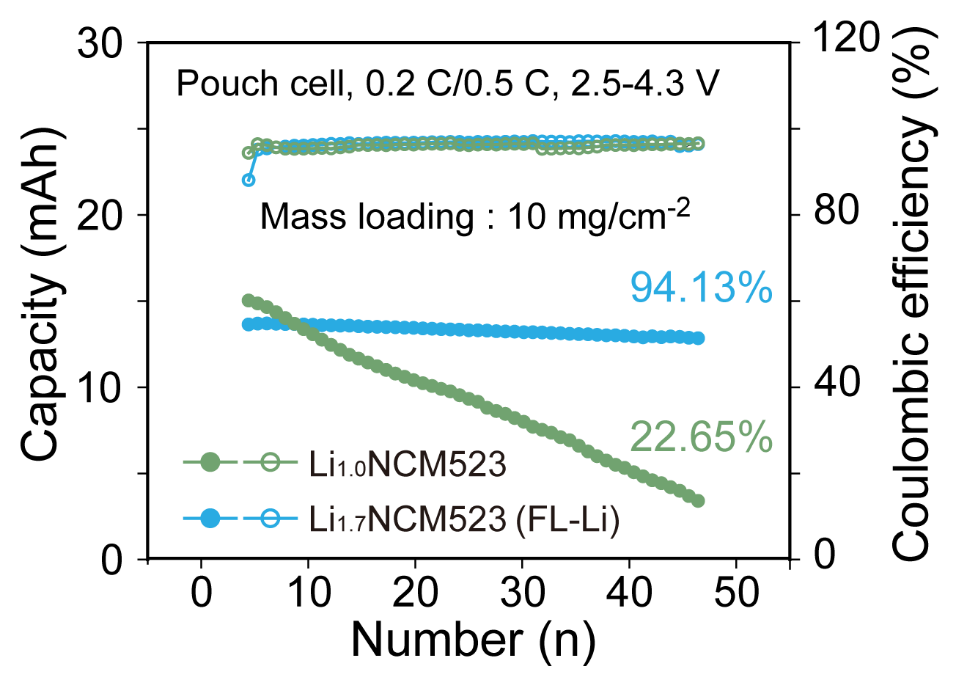


**Figure** **S24**: Cycling stability of Cu||Li_1.0_NCM523 and Cu||Li_1.7_NCM523 (FL-Li) pouch cells under 0.2 C charge / 0.5 C discharge conditions.


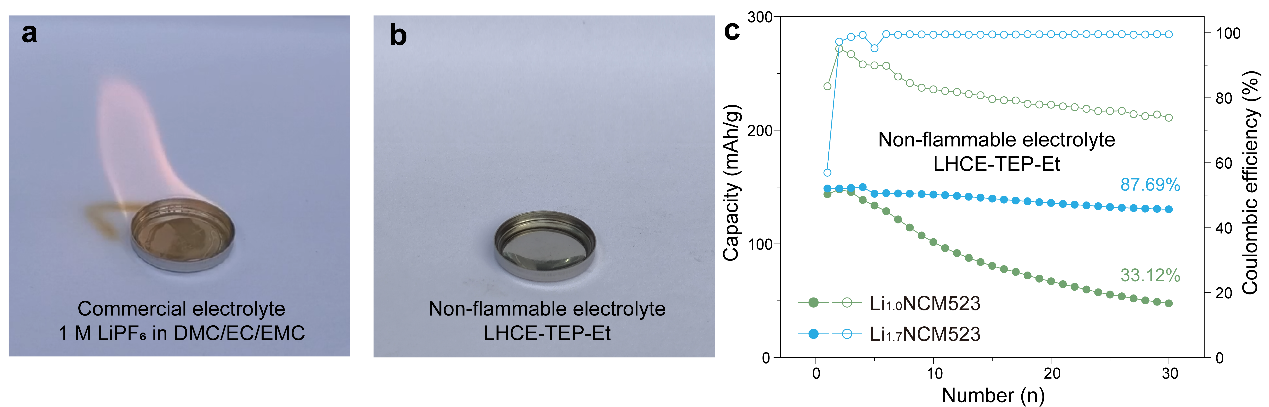


**Figure** **S25**: a-b) Flame test on commercial electrolytes and LHCE-TEP-Et. c) The cycling ability and coulombic efficiency of Cu||Li_1.0_NCM523 and Cu||Li_1.7_NCM523 using non-flammable electrolyte at 0.5 C.

We have included relevant tests for the AFLMBs paired with a non-flammable electrolyte, which composition is 1.27 M of LiFSI dissolved in TEP, DME and TTE, with the ratio as 21:4:75 v:v:v % (LHCE-TEP-Et). We conducted a flame test on commercial electrolytes and LHCE-TEP-Et. The commercial electrolyte ignites immediately upon contact with a flame torch and continues to burn until fully consumed (Figure S24a). However, the TEP-based LHCE-TEP-Et never produced flames, indicating that they are non-flammable (Figure S24b). Cu||Li_1.0_NCM523 (LHCE-TEP-Et) exhibited rapid capacity decay, while Cu||Li_1.7_NCM523 (LHCE-TEP-Et) still had a high capacity retention rate of 87.69% after 30 cycles (Figure S24c), which proves that Li_1.7_NCM523 is very suitable for this high-safety electrolytes.


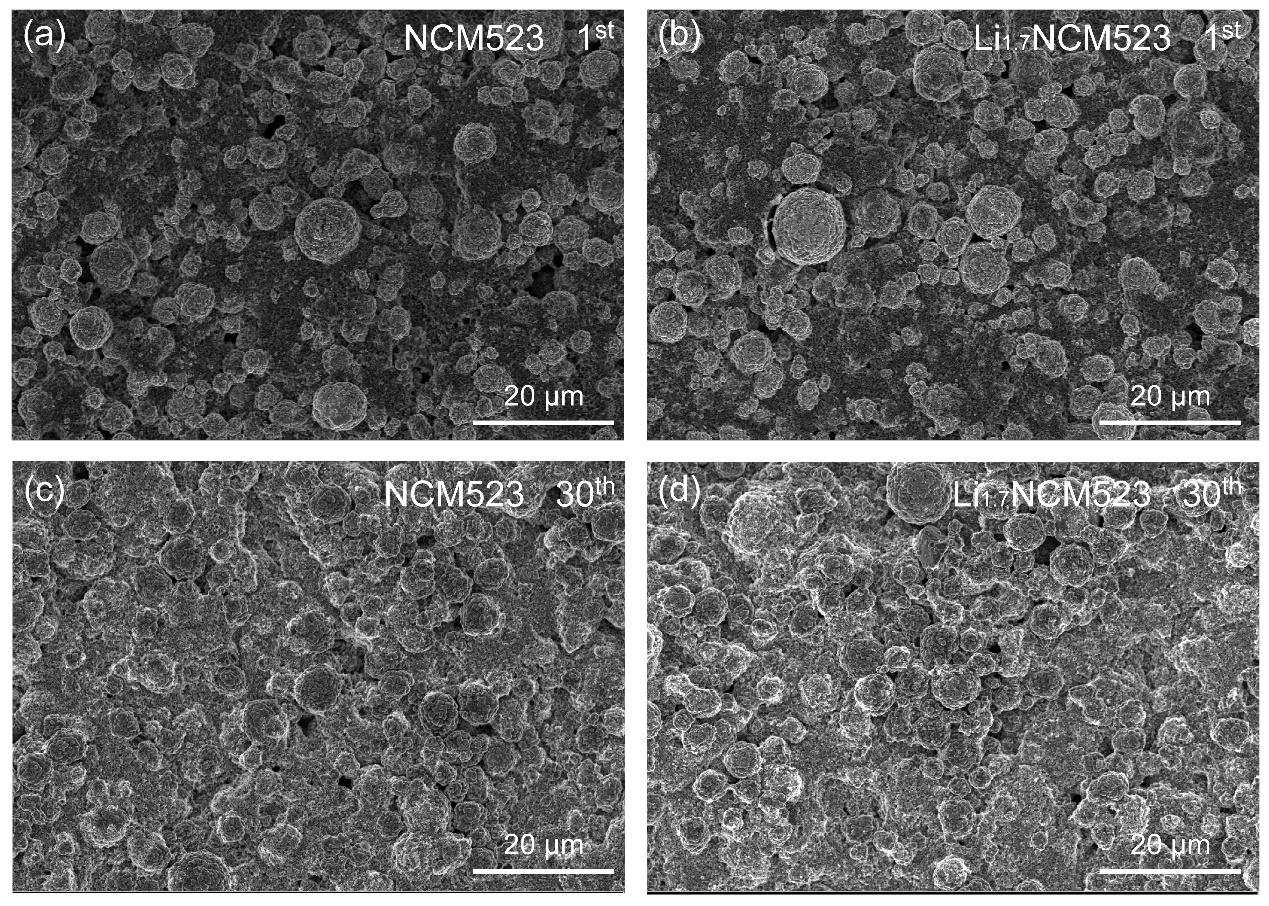


**Figure** **S26**: The morphology changes of the positive electrode of Cu||Li_1.0_NCM523 at a) 1^st^ and c) 30^th^; and Cu||Li_1.7_NCM523 at b) 1^st^ and d) 30^th^.

**
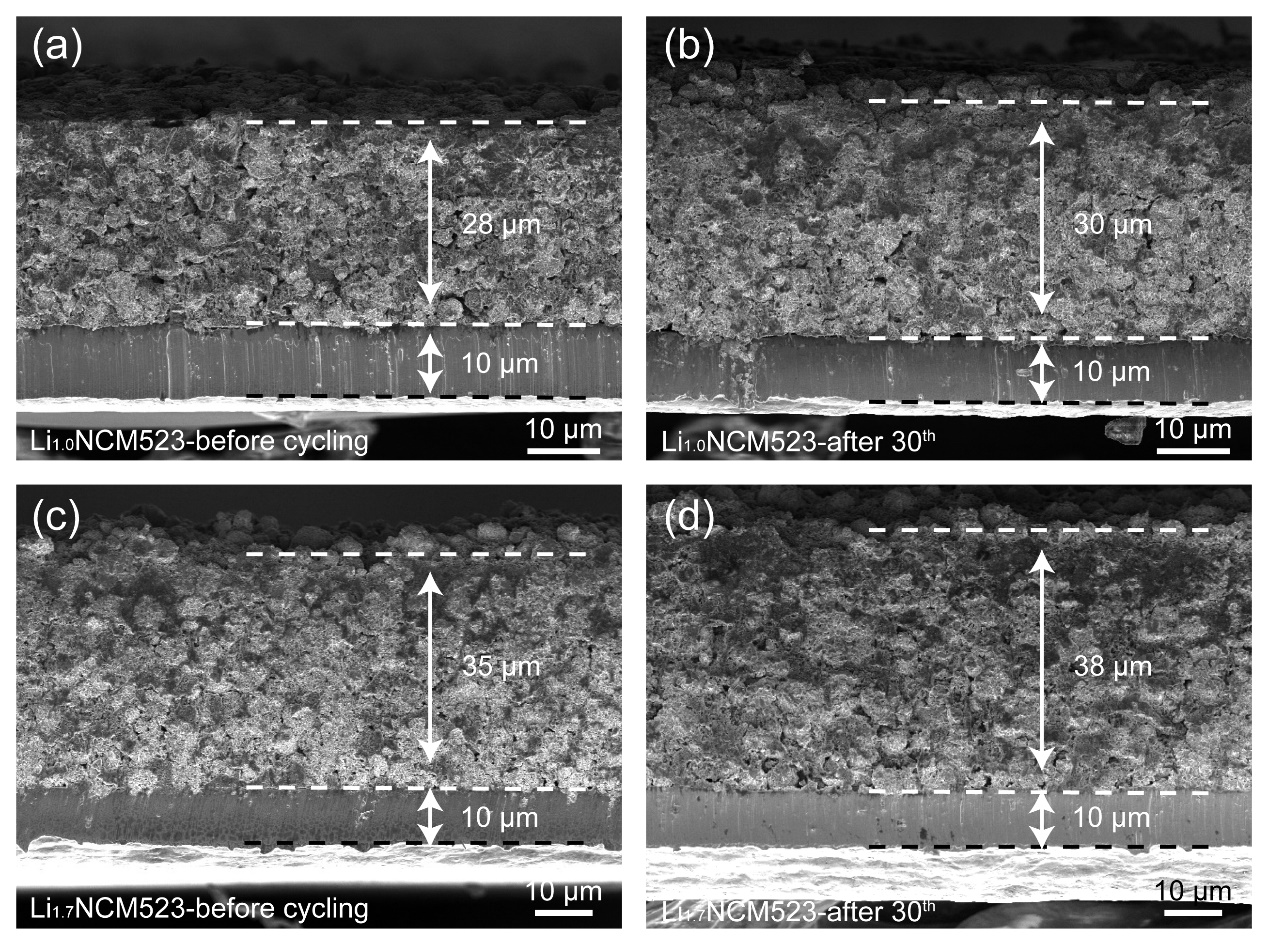
**

**Figure** **S27**: The cross-sectional SEM images of (a) pristine Li_1.0_NCM523, (b) Li_1.0_NCM523-30^th^, (c) pristine Li_1.7_NCM523 and (d) Li_1.7_NCM523-30^th^.

Upon cycling, both Li_1.0_NCM523 and Li_1.7_NCM523 showed minor volumetric expansion, with thicknesses increasing from 28 µm to 30 µm and from 35 µm to 38 µm, respectively. This expansion can be attributed to the mechanical stresses induced in the lattice structure by the repeated intercalation/deintercalation of Li^+^ ions during charge/discharge process. As cycling continues, these stresses accumulate incrementally, finally leading to lattice expansion. Notably, the degree of expansion observed in Li_1.7_NCM523 closely matches that of Li_1.0_NCM523, suggesting that the pre-lithiation process does not compromise the structural stability of cathode.

**
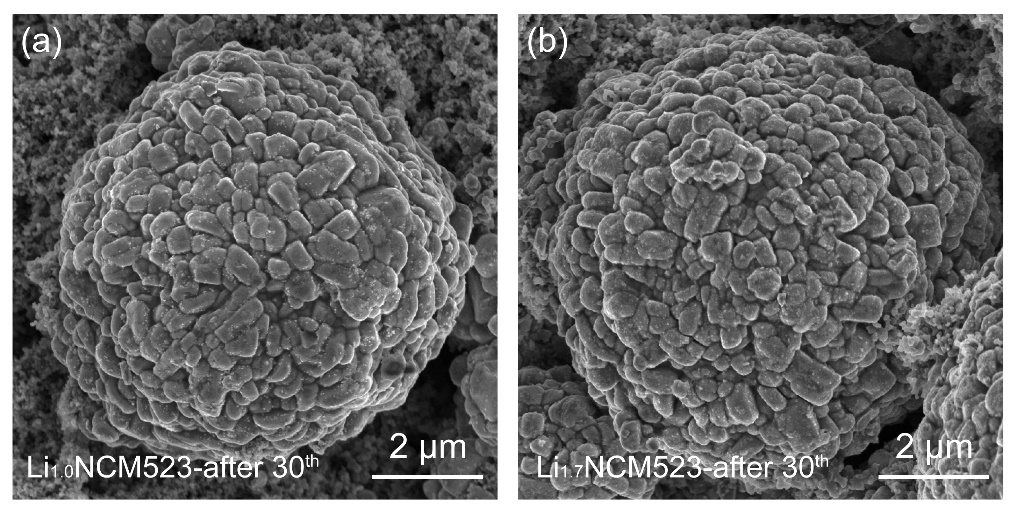
**

**Figure** **S28**: The SEM images of (a) Li_1.0_NCM523-30^th^ and (b) Li_1.7_NCM523-30^th^ single particles.

The SEM images of individual Li_1.0_NCM523-30^th^ and Li_1.7_NCM523-30^th^ cathode particles. Both samples maintained an intact spheroidal morphology, with a uniform CEI layer covering the surface and no visible cracking observed.

**
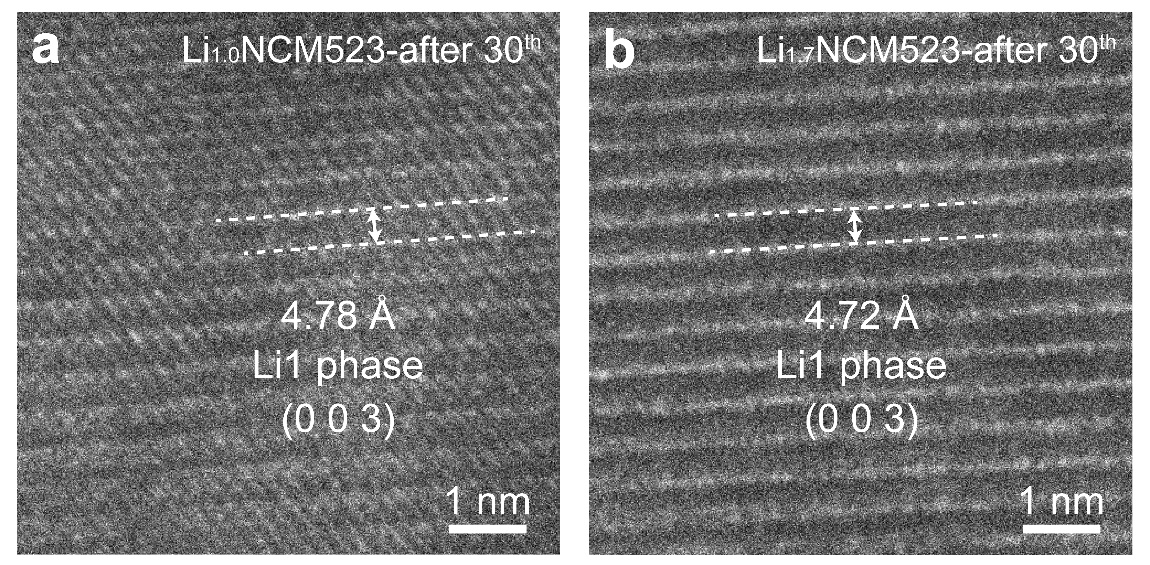
**

**Figure** **S29**: The HR-TEM images of (a) Li_1.0_NCM523-30^th^ and (b) Li_1.7_NCM523-30^th^.

The HR-TEM images of Li_1.0_NCM523-30^th^ and Li_1.7_NCM523-30^th^. The (003) crystal plane of Li_1.0_NCM523-30th exhibits a slight expansion of the lattice spacing to 4.78 Å, indicating that it transforms into a Li-poor state after cycling

**
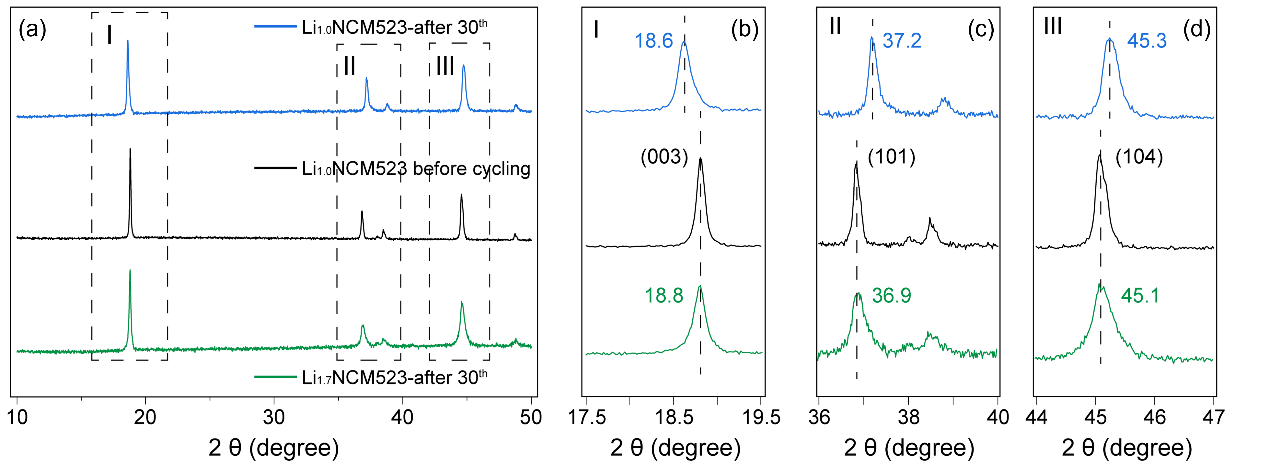
**

**Figure** **S30**: The XRD patterns of (a) pristine Li_1.0_NCM523, Li_1.0_NCM523-30^th^ and Li_1.7_NCM523-30^th^ (b-d) Magnified local regions in Area I (17.5°to 20.5°), Area II (36°to 40°), and Area III (43.5°to 46.5°) of these three materials.

As shown in Figure S29, we tested the diffraction peaks of Li_1.0_NCM523 before cycling, as well as those of Li_1.0_NCM523-30^th^ and Li_1.7_NCM523-30^th^ in the range of 10° to 50°. We then compared their magnified local regions in Area I (17.5° to 20.5°), Area II (36° to 40°), and Area III (43.5° to 46.5°). From Area I, we observed that the (003) peak of Li_1.0_NCM523-30th shifted to a lower angle (0.2°), referring to the expansion in the c-axis. This is due to the continuous lithium loss in Li_1.0_NCM523-30^th^ during cycling, which causes the material transiting to lithium depletion state. As Li^+^ decreases, the electrostatic repulsion between the transition metal layers increases, causing the interlayer space increase. In Areas II and III, we also observed that the (101), (006)/(012), and (104) characteristic peaks of Li_1.0_NCM523-30^th^ shifted towards higher angles, specifically to 37.2° and 45.3°. This is because the average valence states of the transition metals increase in Li_1.0_NCM523-30^th^, as it is in lithium depletion state comparing to Li_1.0_NCM523 before cycling. This causes a shortening of the TM-O bond length (reducing the a and b axes), thereby shifting the corresponding characteristic peaks to higher angles. On the other hand, Li_1.7_NCM523-30^th^, due to the presence of the lithium storage layer, showed no shift in peak positions after cycling. These strongly confirm the advantage of cathode pre-lithiation method.


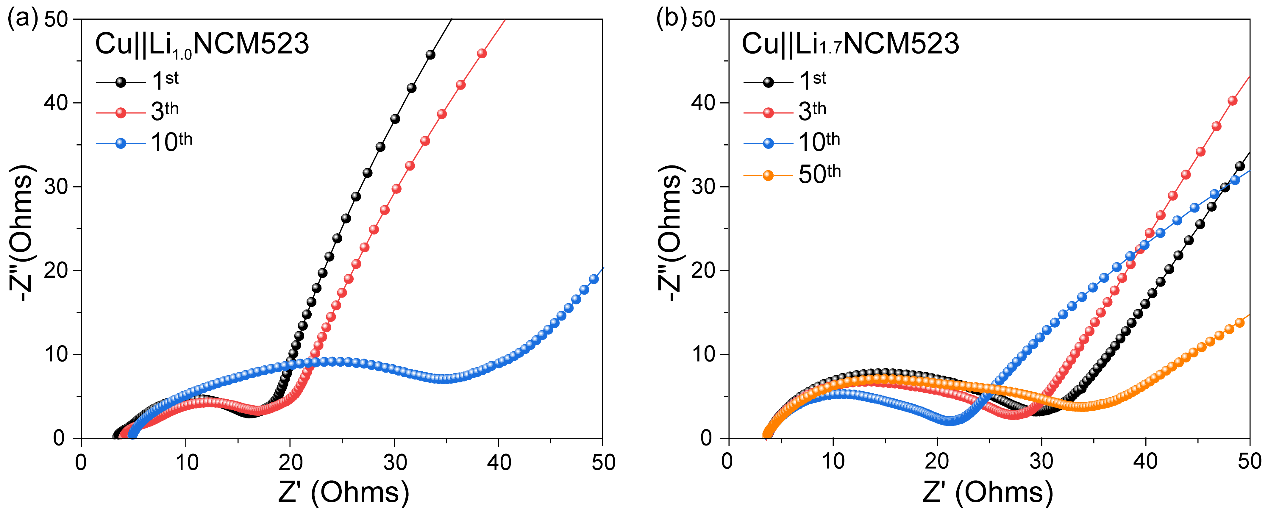


**Figure** **S31**: The EIS test of a) Cu||Li_1.0_NCM523 and b) Cu||Li_1.7_NCM523 during cycling.

**Table 1**：Comparison of our Cu||Li_1.7_NCM523 AFLMBs with other published studies.

| Ref. | Cathode | Optimization strategies | Current density | Cycling number | | Capacity retention | Battery type |
| --- | --- | --- | --- | --- | --- | --- | --- |
| ^[12]^ | NCM811 | * Li_1.35_NCM811 | 1 mA cm^-2^ | 100 | 84% | | pouch |
| ^[13]^ | NCM333 | # Cu\|GPE | 0.5 mAcm^-2^ | 80 | 40.16% | | button |
| ^[14]^ | NCM622 | # [P_1222_][FSI] ionic liquid electrolyte | 1.25 C | 50 | 67.56% | | button |
| ^[15]^ | NCM333 | # Cu\|Ag@PDA-GO | 0.5 mAcm^-2^ | 60 | 55.7% | | button |
| ^[16]^ | NCM811 | † 1 M LiFSI/6FDMH-DME | 0.5 mAcm^-2^ | 100 | 80% | | pouch |
| ^[17]^ | NCM622 | † 4.6 M LiFSI + 2.3 M LiTFSI-DME | 0.33 C | 50 | 60.61% | | button |
| ^[18]^ | NCM333 | # Cu\|GO | 0.5 mA cm^-2^ | 50 | 44% | | button |
| ^[19]^ | NCM622 | * 4-Fluoro-1,2-dihydroxybenzene Li salt | 0.1 C | 100 | 78% | | pouch |
| ^[20]^ | NCM523 | † 1 M LiFSI/FDMB | 0.3 C | 100 | 80% | | Pouch |
|  | NCM622 |  |  |  | 71% | |  |
|  | NCM811 |  |  |  | 70.5% | |  |
| ^[21]^ | NCM811 | # PI@Au | 0.2 C | 100 | 86% | | pouch |
| ^[22]^ | NCM333 | * Li_2_C_2_O_4_ | 0.1 C | 50 | 80% | | button |
| ^[23]^ | NCM811 | * LiNO_3_ | 0.5 C | 50 | 53.03% | | button |
| Our work | NCM523 | * Li_1.7_NCM523 | 1 C | 100  200 | 95.33%  78.45% | | button |

†: Functionalized electrolyte;

#: Modified deposition substrate;

***: Cathode Li compensation agent/Li-rich cathode.

**References:**

[1] G. Kresse, J. Furthmüller, *Computational Materials Science* **1996**, 6, 15.

[2] P. E. Blochl, *Phys Rev B Condens Matter* **1994**, 50, 17953.

[3] G. Kresse, D. Joubert, *Physical Review B* **1999**, 59, 1758.

[4] S. K. Mishra, G. Ceder, *Physical Review B* **1999**, 59, 6120.

[5] J. P. Perdew, K. Burke, M. Ernzerhof, *Physical Review Letters* **1996**, 77, 3865.

[6] V. I. Anisimov, J. Zaanen, O. K. Andersen, *Physical Review B* **1991**, 44, 943.

[7] S. L. Dudarev, G. A. Botton, S. Y. Savrasov, C. J. Humphreys, A. P. Sutton, *Physical Review B* **1998**, 57, 1505.

[8] L. Wang, T. Maxisch, G. Ceder, *Physical Review B* **2006**, 73, 195107.

[9] A. Jain, S. P. Ong, G. Hautier, W. Chen, W. D. Richards, S. Dacek, S. Cholia, D. Gunter, D. Skinner, G. Ceder, K. A. Persson, *APL Materials* **2013**, 1, 011002.

[10] R. Dronskowski, P. E. Bloechl, *The Journal of Physical Chemistry* **1993**, 97, 8617.

[11] S. Maintz, V. L. Deringer, A. L. Tchougreeff, R. Dronskowski, *J Comput Chem* **2016**, 37, 1030.

[12] L. Lin, K. Qin, Q. Zhang, L. Gu, L. Suo, Y. S. Hu, H. Li, X. Huang, L. Chen, *Angew Chem Int Ed Engl* **2021**, 60, 8289.

[13] Y. Nikodimos, W. N. Su, K. N. Shitaw, S. K. Jiang, L. H. Abrha, M. A. Weret, S. K. Merso, T. M. Hagos, C. J. Huang, K. Lakshmanan, W. H. Huang, C. Y. Chang, J. M. Lin, S. H. Wu, C. C. Yang, B. J. Hwang, *Energy Storage Materials* **2023**, 61, 102861.

[14] T. Pathirana, D. A. Rakov, F. Chen, M. Forsyth, R. Kerr, P. C. Howlett, *ACS Applied Energy Materials* **2021**, 4, 6399.

[15] Z. T. Wondimkun, W. A. Tegegne, J. Shi Kai, C. J. Huang, N. A. Sahalie, M. A. Weret, J. Y. Hsu, P. L. Hsieh, Y. S. Huang, S. H. Wu, W. N. Su, B. J. Hwang, *Energy Storage Materials* **2021**, 35, 334.

[16] H. Wang, Z. Yu, X. Kong, W. Huang, Z. Zhang, D. G. Mackanic, X. Huang, J. Qin, Z. Bao, Y. Cui, *Adv Mater* **2021**, 33, e2008619.

[17] J. Alvarado, M. A. Schroeder, T. P. Pollard, X. Wang, J. Z. Lee, M. Zhang, T. Wynn, M. Ding, O. Borodin, Y. S. Meng, K. Xu, *Energy & Environmental Science* **2019**, 12, 780.

[18] Z. T. Wondimkun, T. T. Beyene, M. A. Weret, N. A. Sahalie, C. J. Huang, B. Thirumalraj, B. A. Jote, D. Wang, W. N. Su, C. H. Wang, G. Brunklaus, M. Winter, B. J. Hwang, *Journal of Power Sources* **2020**, 450, 227589.

[19] W. Wu, A. Wang, Q. Zhan, Z. Hu, W. Tang, L. Zhang, J. Luo, *Small* **2023**, 19, e2301737.

[20] Z. Yu, H. Wang, X. Kong, W. Huang, Y. Tsao, D. G. Mackanic, K. Wang, X. Wang, W. Huang, S. Choudhury, Y. Zheng, C. V. Amanchukwu, S. T. Hung, Y. Ma, E. G. Lomeli, J. Qin, Y. Cui, Z. Bao, *Nature Energy* **2020**, 5, 526.

[21] W. Wu, D. Ning, J. Zhang, G. Liu, L. Zeng, H. Yao, M. Wang, L. Deng, L. Yao, *Energy Storage Materials* **2023**, 63, 102974.

[22] C. J. Huang, Y. C. Hsu, K. N. Shitaw, Y. J. Siao, S. H. Wu, C. H. Wang, W. N. Su, B. J. Hwang, *ACS Appl Mater Interfaces* **2022**, 14, 26724.

[23] B. A. Jote, K. N. Shitaw, M. A. Weret, S. C. Yang, C. J. Huang, C. H. Wang, Y. T. Weng, S. H. Wu, W. N. Su, B. J. Hwang, *Journal of Power Sources* **2022**, 532, 231303.
